# Machine Learning Analysis of Enhanced Biodegradable *Phoenix dactylifera L.*/HDPE Composite Thermograms

**DOI:** 10.3390/polym16111515

**Published:** 2024-05-27

**Authors:** Zaid Abdulhamid Alhulaybi, Abdulrazak Jinadu Otaru

**Affiliations:** Chemical Engineering Department, King Faisal University, P.O. Box 380, Al Ahsa 31982, Saudi Arabia

**Keywords:** *Phoenix dactylifera L.*, high-density polyethylene, TGA analysis, machine learning

## Abstract

Worldwide, environmental groups and policymakers are focusing on waste recycling to create economic value and on the decomposition of waste by leveraging on scarce resources. This work, therefore, explores the thermal decomposition of enhanced biodegradable polymer matrices made from a mixture of discarded *Phoenix dactylifera L*./high-density polyethylene (PD/HDPE) using the machine learning analysis of experimental data. The experimental results of these samples were obtained via thermogravimetric (TGA) analysis under an oxidation–free environment, with heating rates of 10, 20, and 40 °C·min^−1^ and a degradation temperature range from 25 to 600 °C. The TGA analyses revealed the continued dependence of the actual percentage weight loss by these materials as a test function of the degradation temperature, shifting thermograms to temperature maxima consistent with increasing heating rates. Although high-density polyethylene (HDPE) materials were found to be thermally more stable than *Phoenix dactylifera L.* (PD) materials, PD/HDPE composite materials contained a significant amount of residual ash. Using a machine learning deep neural network approach for this process, significantly improved learning algorithms have been developed, which reduces the overall cost function (residual error) to almost zero (0.025) after just over a million iterations (epochs) and provides predictions that overlap with the experimental results ($R^{2}\sim 1$). Learning algorithms, along with optimized synaptic weights and biases, were employed to predict the behaviour of PD materials based on experimental thermograms conducted at higher degradation temperatures, typically ranging between 600 and 1000 °C. Predicted data using the enhanced learning algorithms completely overlapped the experiments ($R^{2}\sim 1$) for these higher degradation temperatures with near unity correlation if the decomposition of the materials continued until the residue was attained. With this approach, it is possible to predict and optimize the thermal characteristics of PD and HDPE with greater efficiency, which reduces the need for multiple design iterations and experimentation.

## 1. Introduction

As part of an ambitious plan for Saudi Arabia’s future (Saudi Vision 2030), the Kingdom has taken a circular economy approach to protect the environment and has developed a concept of waste management systems that involves finding sustainable and economic methods for converting, reducing, recycling, and reusing waste. Saudi Arabia’s strategic waste management plan is in line with the 2030 United Nations Sustainable Development Goals, which target waste reduction and reuse (Goal 12) and protecting lives on land (Goal 15) and the marine environment (Goal 14). The 28th meeting of the United Nations Climate Change Conference of Parties (COP 28), held in Dubai, United Arab Emirates, in 2023, included the climate impact of plastic production as part of its agenda that was deliberated. The conference (COP 28) emphasized the importance of ensuring the adequate utilization, reuse, recycling of resources, and safe disposal of any remaining waste. These measures are imperative for the protection of the ecosystem, human health, and the environment. As a result, environmental engineers and scientists face the challenging task of making use of available and limited resources to combat the potential threat of solid waste. It is imperative to reduce the risks associated with solid waste by converting it into more useful products (creating wealth from waste) or providing efficient disposal processes.

High-density polyethylene (HDPE) is one of the most versatile plastic materials that are found in homes, shops, schools, cinemas, and auditoriums, as well as in many industries. These materials are considered thermoplastic polymers [1] and are made from petroleum (petrochemical plants). They are known for their outstanding characteristics of high tensile strength, high melting point and impact resistance, chemical resistance, and high strength-to-density ratios [2]. The combination of these unique characteristics has enabled their wide application in many fields such as plastic bottles, food packaging, cutting boards, pipping, shoe lasts, skeletal and facial reconstruction (plastic surgery), etc. [3,4,5]. Environmental groups and policymakers are concerned about the growing volume of used HDPE materials in the environment. A traditional method for thermally treating waste (burning) often releases air pollutants and contributes to greenhouse gas emissions [6]. Unattended, these materials end up in the marine environment, harming aquatic life. Thermochemical decomposition of these materials, known as pyrolysis, has been used for over a decade as an alternative disposal strategy. This is achieved by heating polymer materials in an oxygen-free environment (inert) which converts the waste into valuable products of oil, syngas, char, or industrial feedstocks [7,8], contributing to energy security and reducing potential air pollution by implementing a circular economy.

Pyrolysis holds immense promise for the degradation of polymer materials, but it is energy intensive. Thus, it is essential to continue research on a more reliable and economical method of reducing energy usage for degradation of polymer materials through its alteration during processing, while keeping in mind the bearing consequences of changes to product quality and process safety as well. To address these limitations and enhance the effectiveness of the pyrolysis process, analogous researchers are actively investigating the possible use of available and sustainable materials as catalysts that reduce the energy required for the thermal degradation of polymer/blend materials. Clays, zeolites, cornplast, biochar, and granite dust, among others, are examples of catalysts that have demonstrated the potential to significantly improve both the efficiency and selectivity of HDPE polymer-making [9,10]. As an example, Awad et al. [11] studied the thermal and mechanical properties of HDPE materials blended with marble and granite dust. Blended materials showed improved mechanical strength, an increase in degradation temperature (11 °C), and a reduction in the loss rate for HDPE of 16.71% in a minute based on thermophysical characteristics. Al-Bayaty et al. [12] studied the thermal degradation of pure HDPE materials at three different heating rates of 10, 15, and 20 °C·min^−1^. The activation energy and reactions models were estimated based on the TGA data using the Coats–Redfern (CR), Flynn–Wall–Ozawa (FWO) iso-conventional, and the Kissinger reaction models.

Contact–Rodrigo et al. [13] studied the thermal degradation of pure HDPE 10062 and copolymer matrices made from 50/50 weight percent of the HDPE material and different additives (Mate—Bi AF05H, Cornplast and Bioefect 72000) for degradation temperatures ranging between 25 and 600 °C. Their thermograms report in [13] showed that the composite materials were less thermally stable in comparison to the pure HDPE 10062 material. Although copolymer matrices showed degradation at lower temperatures, their resulting residues were significantly higher than those produced by pure HDPE 10062 material. Chowdhury and Wang [14] investigated the thermal degradation of polyethylene terephthalate microplastics using kinetics and artificial neural networks (ANN) models at 10, 20, and 30 °C·min^−1^. The ANN modelling approach revealed an optimum two-layer hidden activation which almost overlapped the experimental thermograms with a regression coefficient between 0.95 and 1.00, while the kinetics approach provided estimated activation energies for the materials. A study of recycled copolymer matrices made from extrusion moulding of HDPE material reinforced with bamboo fibre was reported in [15]. This study showed high thermal stability with 10% bamboo fibre loading and an onset of degradation at 362.4 °C, encouraging the use of alternative light-bearing materials in automotive indoor components. Kim et al. [16] investigated the thermal stability of pure HDPE and its composites with rice husk flour (RHF). According to their TGA data [16], the onset of degradation increased to temperature maxima as the heating rate increased from 2 to 40 °C·min^−1^. Further, it was found that increasing the loading of RHF up to 40% into the copolymer matrix resulted in thermograms that completely drove towards temperature minima (decreased thermal stability) but with an increase in ash content.

Although numerous thermogravimetric studies have been conducted on HDPE polymer materials and their composites with either naturally occurring or synthetically produced additives, most of the research has aimed to reduce the thermal degradation of copolymer materials when heated between 10 and 80 °C·min^−1^. Date seeds—or *Phoenix dactylifera L.* (date pits or simply PD)—are an essential part of date fruit, weighing approximately 6–20 percent of the total weight depending on the variety, grade, and maturity [17,18]. However, date fruit production and related process industries generate a lot of waste constituted by a significant amount of these discarded inedible seeds. The objective of this study is to investigate the potential feasibility of employing the machine learning approach of the deep neural networks (DNN) in machine learning to train experimental data related to the pyrolysis of composites consisting of PD and discarded HDPE. The experimental analysis involved the TGA of pure PD and HDPE materials and composites incorporating different ratios of the two materials. Using the machine learning approach, learning algorithms were developed to be applied in the training of experimental data as a function of degradation temperature, time, and heating rate of 10, 20, and 40 °C·min^−1^. Even though the motivation for this project is to contribute to Saudi Vision 2030 by fostering environmental sustainability and resource utilization, it is crucial to note that this study is necessary due to the abundance of PD materials available throughout the Kingdom of Saudi Arabia because of date cultivation throughout the country and other parts of the Middle East region.

## 2. Experimental TGA and Data

In this study, HDPE waste and PD discarded materials and composite matrices of both materials were studied through thermal decomposition. This study follows two stages: preparation of sample and testing with TGA. Samples of HDPE and discarded PD materials were carefully collected, meticulously cleaned, and dried overnight at a temperature of 50 °C, followed by the size reduction of both materials to tiny powders between 100 and 200 µm. An important component of the experiment involved varying the additive-to-polymer mass ratio of PD/HDPE in different matrixes (0/1, 1/3, 1/2, 2/3 and 1/0) before performing TGA analysis. The thermogravimetric (TGA) analysis of these materials was carried out as described in [19]. The thermal stability of these materials was assessed using a Mettler Toledo TGA/SDTA851e device equipped with STARe V7.01 software while heating the materials in an inert environment and increasing the heating rate by 10, 20, and 40 °C·min^−1^. To avoid material loss due to oxidation, the formulated biopolymer matrices were each placed on the sample pan furnace of the TGA device following the purging of nitrogen gas at a rate of 40 mL.min^−1^. Throughout the experimental process, initial measurements of the sample weight were taken wearing gloves to prevent contamination. Initially, the HDPE materials experimented at 10, 20, and 40 °C·min^−1^ weighed 11.83, 11.79, and 12.41 mg, respectively. PD materials experimentally weighed 10.73, 11.95, and 12.28 mg at these heating rates, while composites weighed between 11.65 and 12.91 mg at the different compositions and heating rates. The composites were separately heated between 25 and 600 °C degradation temperatures, and the PD materials were further heated up to 1000 °C for the three heating rates while keeping records (degradation temperature, actual percentage weight loss, time, and heating rate) and monitoring. The TGA instrument was then turned off as soon as the maximum/desired temperature was reached, and the material and instrument were allowed to cool down to room temperature before the weight measurement took place.

**Figure 1:**
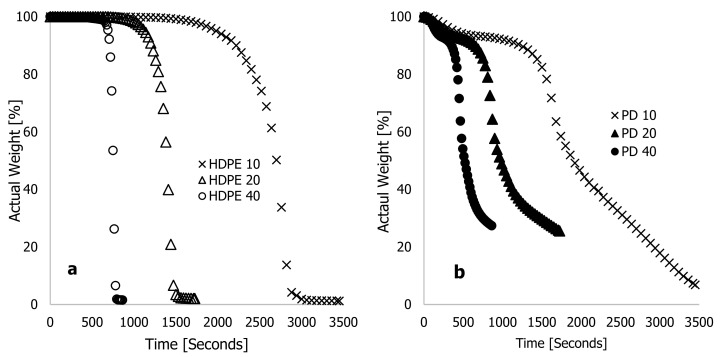
Characteristic thermograms showing typical plots of percentage weight wt % against time (seconds) at different high heating rates of 10, 20, and 40 °C·min^−1^ for (**a**) high-density polyethylene (HDPE) and (**b**) *Phoenix dactylifera L.* (PD) materials.

**Figure 2:**
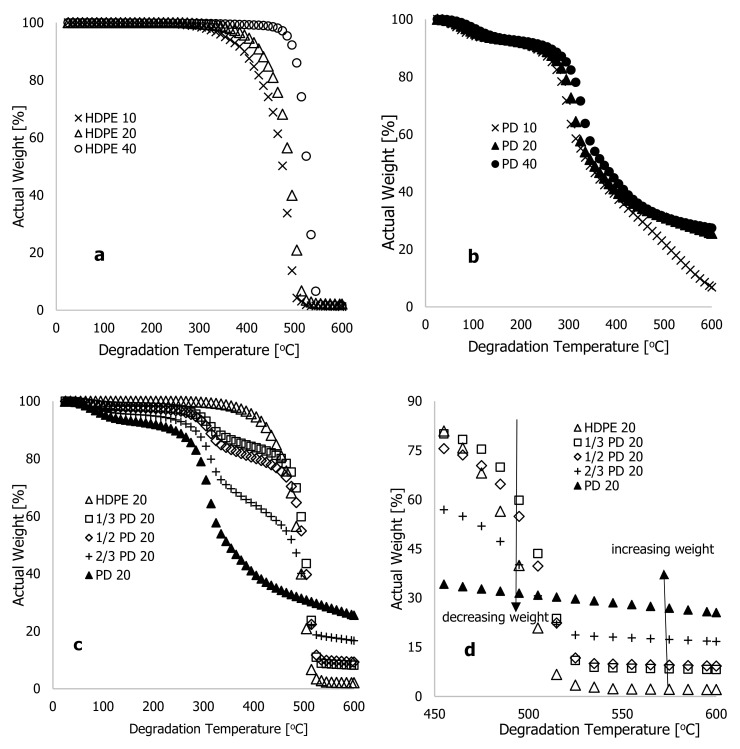
Characteristic thermograms showing typical plots of percentage weight % against the degradation temperature (°C) at different high heating rates of 10, 20, and 40 °C·min^−1^ for (**a**) high-density polyethylene (HDPE) and (**b**) *Phoenix dactylifera L.* (PD) materials; (**c**) mixed composite PD/HDPE for all temperature ranges; and (**d**) mixed composite PD/HDPE from 450 to 600 °C.

Figure 1 shows the actual weight % for HDPE (a) and PD (b) materials against heating time (seconds) for heating rates of 10, 20, and 40 °C·min^−1^. According to these two figures, the heating rate was inversely proportional to the heating time. For the sample run at 40 °C·min^−1^, the recorded completion time was 863 seconds, while the heating time at 20 °C·min^−1^ doubled that of the TGA run at 40 °C·min^−1^, but half of the TGA analysis run at 10 °C·min^−1^. In Figure 2a,b, actual weight % is plotted against degradation temperature (°C) for two materials being heated at 10, 20, and 40 °C·min^−1^ heating rates. There was a general trend of temperature shifting to maxima with increasing heating rate, as substantiated in [16,20,21]. As can be seen in Figure 2a, the actual weight loss of HDPE materials could be divided into three stages: loss of moisture content, decomposition of actual material, and residual content. As the heating rate increased, the onset temperature for a complete loss of moisture content in the materials increased. Accordingly, Figure 2a shows that at 10 °C·min^−1^, HDPE lost 6% of its weight due to moisture loss within a degradation range of 25 to 365 °C. Based on the thermograms conducted at 20 and 40 °C·min^−1^, it was estimated that 3.45 and 4.6% of the actual weight loss had taken place at degradation temperatures of 25–385 °C and 25–485 °C, respectively. The decomposition phase of thermograms contributed significantly to the loss of material content during all heating rates, and the changes occurred instantly with a shrinking range of degradation temperatures with increasing heating rates. Figure 2a shows, for example, that the materials decomposed at a 10 °C·min^−1^ heating rate at a degradation temperature ranging between 365 and 535 °C, resulting in 91 percent material loss. In the same way, at 20 and 40 °C·min^−1^ heating rates, the material content of these samples was lost at degradation temperatures between 385 and 525 °C (93.0%) and between 485 and 555 °C (93.14%), respectively. In this pyrolysis stage, a substantial weight loss was achieved, further increasing as the heating rate, the degradation temperature range, and the time spent in the oxidation-free environment were reduced.

The thermograms in Figure 2b show plots of the actual weight % against the degradation temperature (°C) for PD materials heated at different rates of 10, 20, and 40 °C·min^−1^. There was a similar shift in temperature maxima with increasing heating rate as in Figure 2a; however, the thermogram at 10 °C·min^−1^ diverged from the other two heating rates at 445 °C degradation temperature. Possibly, this was because the sample was heated at 10 °C·min^−1^ for a delayed period, ensuring that materials degraded slowly by consistently decomposing into ash, as well as a change in physiochemical properties and activation energy (recommended for future research on the process’ kinetics). A significant difference was observed in the thermograms of the PD materials compared with the HDPE materials. The moisture content loss in the PD materials was higher than that in the HDPE materials, and a shift to temperature minima was observed at degradation temperatures between 25 and 500 °C. According to Figure 2b, degradation began at low temperatures such as 35 °C, regardless of the heating rate of the PD materials. According to the estimate, 14.44% of actual weight was lost due to moisture content at 10 °C·min^−1^ heating rate (35–275 °C). However, at 20 and 40 °C·min^−1^ heating rates, the actual weight losses were 17 and 17.6%, respectively, with degradation temperatures ranging from 35 to 285 °C and from 35 to 305 °C, respectively. As a result, PD materials had higher moisture content than HDPE materials. After this stage of degradation, the decomposition of PD materials continued up to a maximum degradation temperature of 600 °C, and a shift in temperature maximum in the thermograms was consistent with increased heating rates. 

According to Figure 2b, PD materials weight fractions at higher degradation temperatures, typically above 500 °C, were significantly higher than those for HDPE at all heating rates. From the raw data, the residual weight of HDPE materials at this maximum degradation temperature (600 °C) and for all three heating rates were 0.15, 0.25, and 0.19 mg, respectively. PD materials yielded 0.74, 3.06, and 3.36 mg for the three heating rates and 600 °C maximum degradation temperature, respectively. As shown in Figure 2a, HDPE materials at different heating rates degraded completely to ash, but the trends in Figure 2b indicate that degradation continued beyond this temperature, and at temperatures above 600 °C, the PD materials could be completely degraded (to be discussed later). In Figure 2c,d, the actual weight loss is plotted for HDPE, PD, and composites at 20 °C·min^−1^. As the additive (PD) was added to the polymer matrix (HDPE), the degradation temperature decreased (decreased thermal stability), resulting in a shift to temperature minima for degradation temperatures between 25 and 500 °C. Figure 2d shows that, beyond this degradation temperature of 500 °C, the increasing weight of the residual material increased with the increasing addition of additives in the polymer matrix, resulting in a delay in full degradation, which requires another increase in degradation temperature to fully decompose the PD materials. A study conducted by Kim et al. [16] suggested that incorporating rice husk flour into thermoplastic polymers reduces the thermal stability of the matrix. This resulted in a shift to temperature minima and an increase in ash content as the ratio of rice husk flour increased. According to the current experiment, the thermal stability of the materials decreased with the increasing additives in the polymer matrix; however, this cannot be said for the ash content until PD materials are completely degraded. Further experiments on the degradation temperature of PD materials beyond 600 °C are therefore imperative, and the experimental approach and data are discussed during the modelling phase.

## 3. Machine Learning Algorithms and Data

Modelling this system behaviour using machine learning began with understanding raw thermogram data obtained from individual and mixed composite samples and categorizing them into input and output signals. In this case, the actual or experimental percentage weight % loss from thermograms served as the output signal, which was affected by the degradation temperature (T), time (t), and heating rate (Q). For all samples at different heating rates, Figure 1 and Figure 2 show that the thermograms were non-linear inverse with increasing degradation temperature, indicating the need for the formulation of learning algorithms for the experimental data to be trained using deep neural networks (DNN). A non-linear relationship between input and output data from a system can be more reliable when using deep neural networks, as reported in [22,23,24]. Its convolution tends to improve the process as well as reduce the difference between predicted and real data errors. For simple linear relationships between input signals (independent variables) and output signals (dependent variables), the single neural network (SNN) is frequently recommended, just like least-squares regression models [25]. 

**Figure 3:**
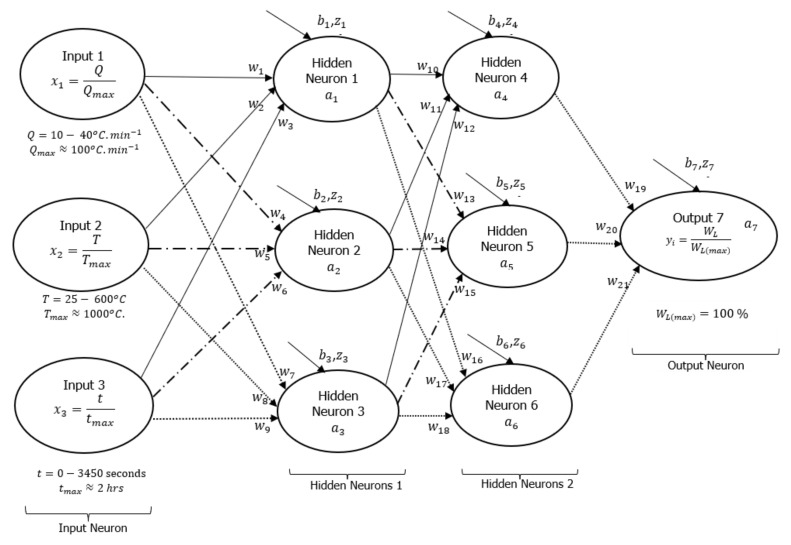
Machine learning DNN framework showing typical three-input (1 layer) neurons, six hidden (two layers) neurons, and an output neuron.

A DNN framework was initially drawn to explain how the input and output signals were interconnected with some hidden layers (neurons) as shown in Figure 3, following a detailed assessment of all experimental data conducted at heating rates of 10, 20, and 40 °C·min^−1^. Based on this initial guess, there were two layers of six hidden neurons linking each of the three input signals to the output signal (actual weight %). The neurons were connected by 21 synaptic weights ($w_{i}$) and seven biases ($b_{i}$). Sirca et al. [22] referred to these synaptic weights and biases as arbitrary constants that often change during training. Another article by Panneerselvam [24] stated that changes in bias during the training process are a sign that the flexibility in the output response creates an offset. Inputs have an impact on outputs, whereas changes to synaptic weights are an indication of the inputs’ influence. Learning algorithms were developed using artificial neural networks (ANNs) presented in equations [1,2,3], as reported in [22,23]. Equation (1) shows the dependence of the sum weight (Z) as a function of synaptic connections, input signals, and biases, while Equation (2) shows the logistic function of the sum weight (σ′[z]). Mathematically expressed, Equation (3) shows the cost function (C) as the residual error between the real (y) and predicted/activation (a) output signals.

Equation (2) is the Sigmoid-activated function selected for the formulation of the learning algorithms in Figure 3. There are other non-linear activation functions, such as hyperbolic tangents (TanH), rectified linear units (ReLU), exponential linear units (ELU), etc. However, the choice of Sigmoid activation functions is based on their ability to converge data points that are between 0 and 1 based on changes in arbitrary constants during training [20,24,26]. For this reason, the input and output data points were divided by the maximum value possible for a TGA experiment as part of data preparation before training. As shown in Figure 3, the maximum degradation temperature, heating rate, time, and weight loss parameters were 1000 °C, 100 °C·min^−1^, 2 h, and 100%, respectively.

(1) $$z_{j}=\sum_{i}w_{i}.x_{i}+b_{k}$$

(2) $$a_{j}=\sigma \prime [z_{j}]=\frac{1}{1+e^{-z_{j}}}$$

(3) $$C={\left(y-a_{j}\right)}^{2}$$

The primary objective of the machine learning technique used herein is to develop learning algorithms for finding the optimal arbitrary constants (i.e., synaptic weights and biases) necessary to close the gap between prediction and experiment. While learning algorithms are of utmost importance, it is also essential to determine the proportion of datasets that will serve as input signals to the DNN framework. The selected experimental datasets used for training were categorized based on thermograms for different compositions: 100% HDPE, 33.3% PD, 50.0% PD, 66.6% PD, and 100% PD. These thermograms were conducted at heating rates of 10, 20, and 40 °C·min^−1^ for each composition. This approach resulted in a total of 15 datasets (thermograms), with each dataset containing 59 data points. Hence, the total number of experimental data points was 885. The actual raw data generated for these experiments totalled 30,145, obtained at temperature differences of 0.17, 0.33, and 0.67 °C, corresponding to the heating rates of 10, 20, and 40 °C·min^−1^, respectively. Therefore, the 885 data points used for training were selected from the raw thermogram to be their representative, from 25 to 600 °C, with intervals of 25 °C. The remaining data points were reserved for validation and testing.

**Figure 4:**
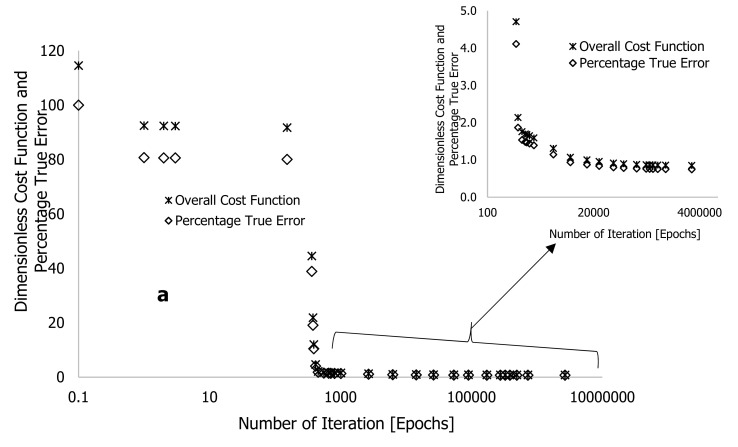

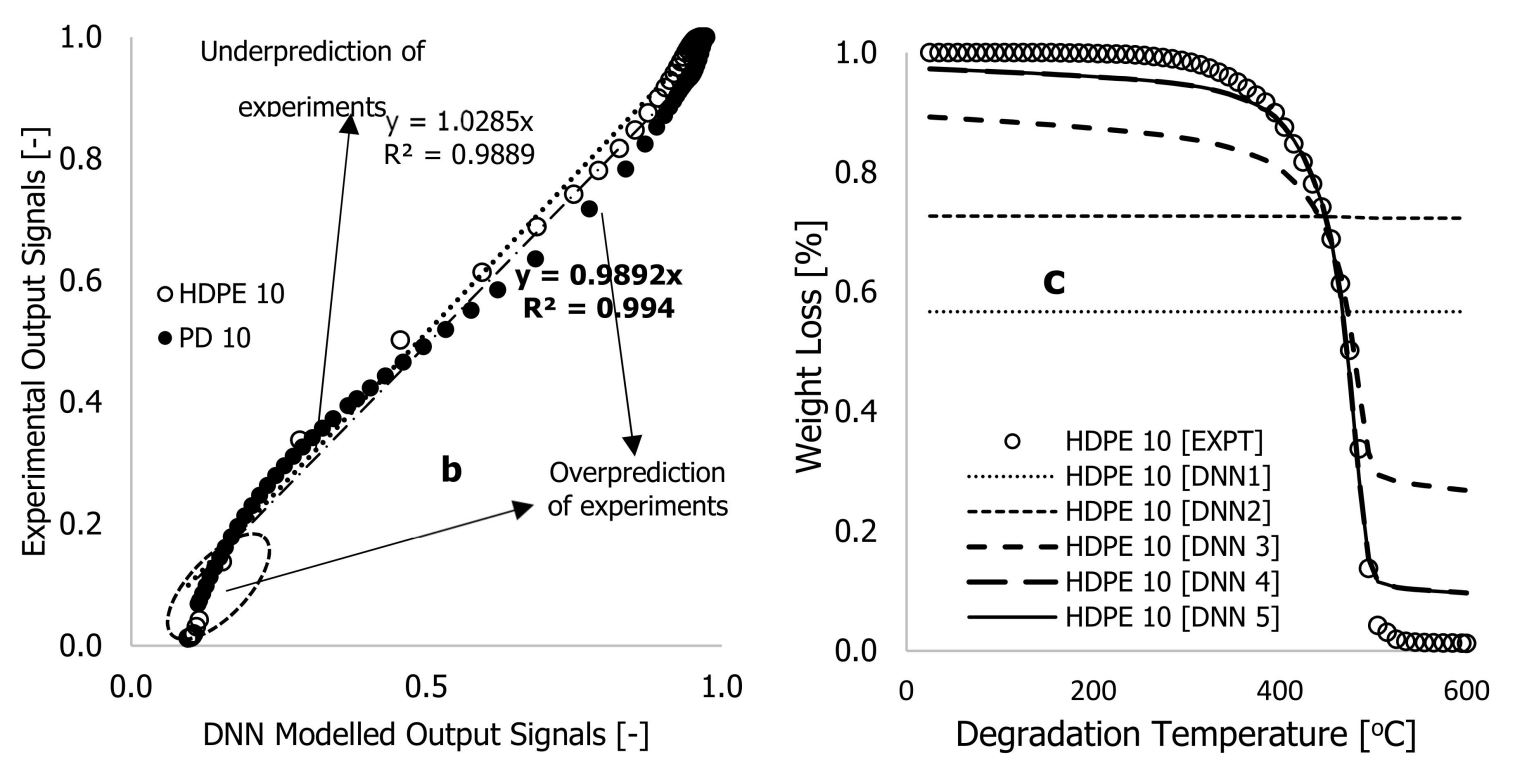
Plots of (**a**) dimensionless cost function and percentage true error against the number of iterations, (**b**) experimental output signals against DNN-modelled output signals, and (**c**) experimental and DNN-modelled weight loss (%) against degradation temperature, using learning algorithms developed from neural network with six hidden neurons and two layers (Figure 3).

The training of this process was performed in Microsoft Excel with code written in its Visual Basic for Applications (VBA). Following the training (simulation) on a computer, the cost function and approximate or true error can be reduced to almost zero. Figure 4 presents the response obtained after training the experimental data using formulated learning algorithms within the DNN framework shown in Figure 3. Table 1 shows the extracted synaptic weights ($w_{i}$), biases ($b_{i}$), linearity rates ($L_{R}$), cost functions ($\sum_{i=1}^{n}C$), number of iterations (epochs or loops), approximate error ($\varepsilon_{a}$), and true error ($\varepsilon_{t}$). The estimation of these errors was determined using equations reported in [25]. The purpose of the training process was to minimize the overall cost function, as well as the true and approximate errors to nearly zero while also reducing the gap between predictions and experiments. As shown in Figure 3, six hidden neurons (also called hyperparameters) were initially selected, which may need to be enhanced after training.

In Table 1 and Figure 4, the overall cost function and true error decreased gradually over the first four iterations with a linearity rate of 5 and 6 hyperparameters following a looping time of one second specified in the written code for the completion of each iteration. At the onset of the training, the true error ($\varepsilon_{t}$) and overall cost function ($\sum_{i=1}^{n}C$) significantly reduced after over 392 iterations (epochs), reducing their values by approximately 12.02 and 10.5%, respectively. The code in MS Excel VBA was then edited to set the looping time to zero. This change increased the processing speed of data changes and enabled the computation of more epochs in a period. Based on Table 1, the simulation process was stopped at 2,688,013 epochs, obtained at a linearity rate of 0.5, with a reduced overall cost function of 0.852, an approximate error of 0.833%, and a true error of 0.744%. This training process was stopped after 149 h (approximately 12 nights), as convergent cost functions and errors may take longer to arrive. It is worth noting that before the completion of the training, the true error ($\varepsilon_{t}$) was reduced from 0.750 to 0.744% after over two million epochs and 10 nights of training time.

**Figure 5:**
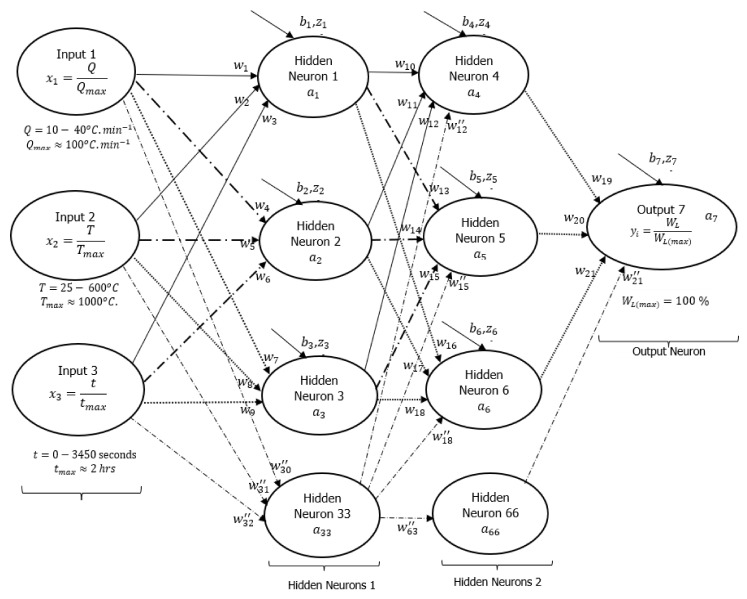
Machine learning DNN framework showing typical three-input (1 layer) neurons, eight hidden (two layers) neurons, and an output neuron.

This point is reinforced by plots of weight loss against degradation temperature of the HDPE thermogram conducted at 10 °C·min^−1^, plotted in Figure 4c. To begin the training, equal values of arbitrary constants (0.1) were selected, and the result was a pattern (DNN1 in Figure 4c) that remained constant as degradation temperature increased or simply trapped in local minima. These arbitrary values changed visibly during the training period as the correlations between DNN modelling and experimentation improved with increasing epochs, as shown in Figure 4b,c. Overall, the correlations between the modelled and experimental data were reasonably within the scatter. However, there were instances of overfitting and underfitting of the finally trained data against some of the experimental data. This is particularly notable in Figure 4b,c, as the final trained data only exhibited complete overlap with the experimental data at the decomposition stage, specifically accounting for the degradation temperature range between 400 and 500 °C in the thermogram. To rectify this discrepancy, it was imperative to minimize the cost function and true error to achieve a precise alignment between predictions and experimental results, specifically concerning the sections of weight loss caused by moisture content (25–380 °C) and residue (500–600 °C) in the TGA data. Given this, it was crucial to consider an alternative method to achieve both convergence and accuracy in a shorter training time, considering the limited resources available. To enhance the training process, an additional two hidden neurons were incorporated into the existing DNN framework, shown in Figure 5, and distributed evenly across the two hidden layers.

**Figure 6:**
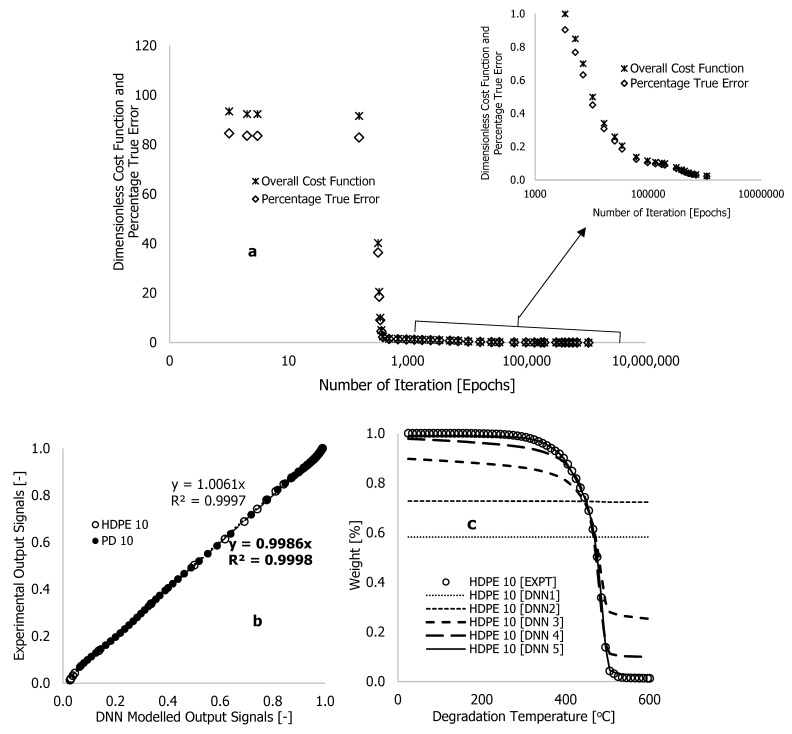
Plots of (**a**) dimensionless cost function and percentage true error against number of iteration, (**b**) experimental output signals against DNN modelled output signals, and (**c**) experiment al and DNN modelled weight loss (%) against the degradation temperature, using learning algorithms developed from a neural network with eight hidden neurons and two layers (Figure 5).

The purpose of adding neurons to the hidden layers is to improve training and was done based on suggestions and findings substantiated in [21,22,23]. Figure 6 illustrates an improvement in training results following training using the enhanced DNN framework in Figure 5. The overall cost function ($\sum_{i=1}^{n}C$) and percentage true error ($\varepsilon_{t}$) were reduced from 110.5 to 0.025 and from 100% to 0.023%, respectively, with short learning epochs and reduced computational time (Table 2). Compared with the originally developed algorithms, Table 2 shows that the enhanced algorithms required a computational time of 60 h (approximately five training nights), 1,090,594 epochs, and linearity rates of 5 and 2 used at the beginning and end of the training process, respectively, resulting in a true error of approximately zero and a cost function of near zero, as shown in Figure 6a. Figure 6b,c illustrate how the formulated learning algorithms led to complete overlap between DNN predictions and experimental outcomes throughout the thermograms. With this approach, overfits and underfits previously associated with the earlier training process were reduced.

**Figure 7:**
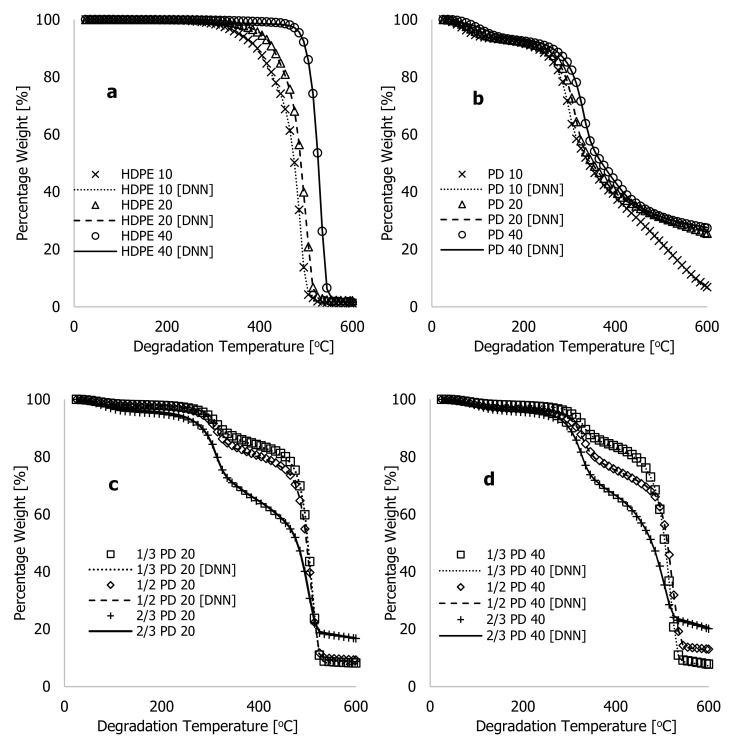
Plots of experimental (actual) and DNN-predicted weight % against the degradation temperture (25–600 °C) for (**a**) HDPE materials at 10, 20, and 40 °C·min^−1^ heating rates; (**b**) PD materials at 10, 20, and 40 °C·min^−1^ heating rates; (**c**) PD/HDPE mixtures at a 20 °C·min^−1^ heating rate; and (**d**) PD/HDPE mixtures at a 40 °C·min^−1^ heating rate.

**Figure 8:**
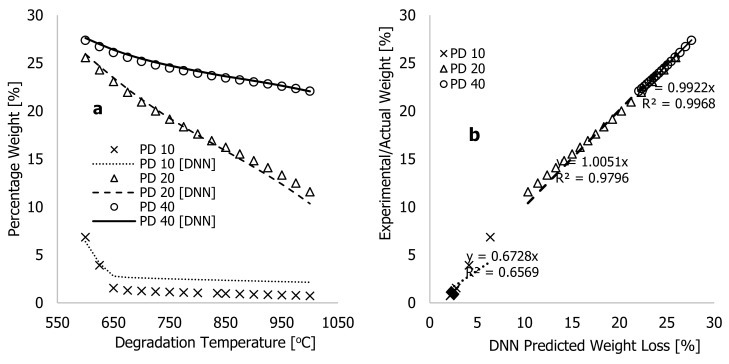
Plots of (**a**) experimental (actual) and DNN-predicted weight % against the degradation temperture (625–1000 °C) and (**b**) plots of experimental (actual) weight loss against DNN-predicted weight loss (%) for PD materials at 10, 20, and 40 °C·min^−1^ heating rates.

Figure 7 shows both the actual (experiment) and DNN-predicted percentage weight loss for HDPE, PD, and composites of both along with degradation temperature (°C) for all the samples. The figures show that the improved learning algorithms accurately described the characteristic thermograms generated from TGA experiments, thus confirming the model’s accuracy. Several experimental TGA data points for PD materials were conducted at higher degradation temperatures, typically between 600 and 1000 °C, and at different heating rates to better understand the limitations of the developed algorithms. These data were obtained during the TGA analyses of the PD materials as the degradation temperature was studied between 25 and 1000 °C at three different heating rates of 10, 20, and 40 °C·min^−1^ before the TGA system being turned off and allowed to cool before samples were removed and weighed. The plots in Figure 8a show experimental data and the DNN models using the much-improved learning algorithms, along with the training/optimised arbitrary constants against degradation temperatures ranging between 600 and 1000 °C. At 10 °C·min^−1^ of heating rate, weight loss by the PD materials between 600 and 650 °C reduced from 6.85 to 1.54%, and beyond this temperature, changes associated with weight loss remained nearly the same. This indicates that the material had completely decomposed at 10 °C·min^−1^. Above this temperature, all that remained was ash or residue. In contrast, Figure 8a illustrates that PD materials continued to decompose between 600 and 1000 °C for heating rates of 20 and 40 °C·min^−1^. The residual value of 1.54 weight % recorded after complete decomposition of PD material at 10 °C·min^−1^ indicates that this material contained more ash than the HDPE despite changes in process conditions during the TGA test. Thus, higher degradation temperatures are required for further breakdown of PD and composite materials at higher heating rates.

The results of Figure 8b show that the new learning algorithms could reasonably predict the behavior for PD materials at 20 and 40 °C·min^−1^ within experimental scatters with estimated correlation coefficients of approximately 1.0. However, the predicting accuracy of the thermograms at 10 °C·min^−1^ heating rates were observably poor beyond the degradation temperature of 650 °C; this might may have been to the fact that complete decomposition of the material occurred much earlier than it did for samples heated at 20 and 40 °C·min^−1^. Accordingly, the much-improved learning algorithms are capable of accurately predicting the thermograms of individual and composite materials up to the residual ash point during the degradation period. In Table 2, biases were observed to change within the training period, creating an offset in the balance and flexibility of the learning algorithms in understanding the experimental thermograms. More importantly, the ongoing changes to synaptic weights ($w_{1}-w_{33}^{\prime \prime }$) during the training period contribute to the effects passed on from preceding neurons to succeeding neurons. According to Panneerselvam [24], a positive value in a trained synaptic weight indicates that the input data directly influences the output signals. Conversely, an increase in negative value in the synaptic weight reduces the likelihood of the input data positively influencing the output signal. Both the negative and positive synaptic weights were recorded during both training processes, creating continuous swings of DNN data. This resulted in the complete overlap of experiments and a significant reduction in the overall cost function and true error, as shown by Table 2. The significant changes related to the biases and synaptic weights of the finally trained learning algorithms, along with the complete overlap of both modelling and experiments, further emphasize the contributory role of the added neurons in optimizing the training process to achieve higher accuracy and quicker convergence.

Reducing this true or residual error to a near-zero level may require an increase in training time or an expansion of the training data capacity, commonly known as data augmentation [24], to mitigate data overfitting. It is important to note that the DNN framework shown in Figure 5 is characterized by eight hidden neurons (HNS), making it a more complex framework compared to the DNN architecture with six hidden neurons (Figure 3). Despite the complexity of the eight HNS framework, the resulting output signals were superior to those of the six HNS framework. To reduce overfitting of the output data in the eight HNS framework, improvements were made to the model capacity limit by adding two active neurons, varying the linearity rate ($L_{R}$) during training, and implementing early stoppage of training to prevent validation losses. These improvements were made while maintaining a similar number of selected data points, avoiding data augmentation to save modelling time. The addition of extra neurons increased the number of weights and biases, gradually decreasing the overall cost function (residual error) in a timely manner with fewer epochs. Furthermore, changes in the linearity rate ($L_{R}$) of the learning algorithms during training were achieved by setting the training data within a range from 5 to 2. Beyond this range, both the synaptic weights and biases increased significantly, leading to increased overfitting and true error (validation loss). Additionally, selecting a learning rate below this range resulted in a significant reduction in both the synaptic weights and biases, resulting in delayed convergence and increased modelling time. Therefore, great care was taken to ensure that the selection of the linearity rate value used in the modelling remained between 5 and 2. Reducing the training time and epochs was crucial for the success of the modelling outcome, and this was achieved by early stoppage of training whenever a divergence occurred and subsequent adjustment of the modelling capacity to achieve optimum synaptic weights and biases capable of minimizing the true error between the modelling and experimental results to nearly zero.

In addition, it is worth noting that the learning algorithms used for training the experimental data for these materials were formulated as test functions of the degradation temperature, heating rate, and time. Although this could assist materials manufacturers in predicting the thermograms of PD and HDPE materials at heating rates between 10 and 40 °C·min^−1^, the same confidence cannot be extended to the composites as their compositions was not included in the learning algorithms. By including their compositions, the process may become overcomplicated and could call for practical implication of the additive-to-polymer ratios on their kinetics and thermodynamics. As such, this team is also studying the kinetics and thermodynamics of such composites and improving DNN learning algorithms.

## 4. Conclusions

This study utilised a machine learning deep neural network technique to develop algorithms for analysing the thermograms of enhanced biodegradable *Phoenix dactylifera L.*/High-Density Polyethylene (PD/HDPE) composites obtained from experimental TGA data. The experimental TGA approach reliably showed the increasing percentage weight loss of these materials over a degradation temperature range from 25 to 600 °C for three different heating rates: 10, 20, and 40 °C·min^−1^. Results from this experimental TGA study strongly support established findings in the literature regarding the consistent shift of thermograms to temperature maxima with an increasing heating rate. The addition of the additive (PD) into the composites consistently decreased their thermal stability due to the high moisture content of the PD material. However, the ash content of the composite noticeably increased with increasing addition of PD material. The formulation of the learning algorithms was useful in training this process, and the DNN-modelled data showed reasonable correlations when compared with the actual thermograms (experiments). Though initially formulated, the algorithms for a two-hidden layer framework consisting of equally distributed hidden three neurons in each hidden layer, linked by several synaptic weights and biases, aptly reduced the overall cost function (residual error) between modelling and experimentation from 114.51 to 0.85. Aside from the longer training period experienced with the application of these formulated learning algorithms, this was notably not enough to completely close the gap between predictions and experiments. The overall cost function was then brought down to nearly zero (~0.025), following the formulation of new learning algorithms for a much-improved DNN framework consisting of two additional and equally distributed neurons in the hidden layers. The improved learning algorithms and optimized arbitrary constants (synaptic weights and biases) were used to predict the behaviour characterized by experimental thermograms of the PD materials conducted at three different heating rates of 10, 20, and 40 °C·min^−1^ and degradation temperatures ranging between 600 and 1000 °C. The predictions made by DNN were accurate within the range of experimental variability until the materials degraded completely and turned into ash or residue. These developed algorithms can potentially support manufacturers in predicting the thermal stability of these composites, thereby aiding in the design of polymer matrices that exhibit optimal operability.

## Figures and Tables

**Table 1:** Tabular representation of trained data using learning algorithms developed for the neural network with six hidden neurons and two layers (Figure 3).

	**b_1_**	**w_1_**	**w_2_**	**w_3_**	**b_2_**	**w_4_**	**w_5_**	**w_6_**	**b_3_**	**w_7_**	**w_8_**	**w_9_**
DNN	−1.8174	0.0325	−0.1819	0.5452	−1.8174	0.0325	−0.1819	0.5452	−1.8174	0.0325	−0.1819	0.5452
	**b_4_**	**w_10_**	**w_11_**	**w_12_**	**b_5_**	**w_13_**	**w_14_**	**w_15_**	**b_6_**	**w_16_**	**w_17_**	**w_18_**
DNN	−2.6144	3.3495	3.3495	3.3495	−2.6144	3.3495	3.3495	3.3495	−2.6144	3.3495	3.3495	3.3495
	**b_7_**	**w_19_**		**w_20_**	**w_21_**	**L_R_**	$\sum_{i=1}^{n}C$		$I_{N}$	$\varepsilon_{a}$		$\varepsilon_{t}$
DNN5	−8.7663	10.3697		10.3697	10.3697	0.5	0.8522		2,688,013	0.833		0.744

NB: b_i_ is the bias, w_i_ is the synaptic weight, L_R_ is the linearity rate, C is the cost function, $I_{N}$ is the number of iterations or loops, $\varepsilon_{a}$ is the approximate error, and $\varepsilon_{t}$ is the true error. Training time (t) = 149 h~12 days.

**Table 2:** Tabular representation of trained numerical data using learning algorithms developed for the neural network with eight hidden neurons and two layers (Figure 5).

	**b_1_**	**w_1_**	**w_2_**	**w_3_**	**b_2_**	**w_4_**		**w_5_**	**w_6_**	**b_3_**	**w_7_**	**w_8_**
DNN	−0.3166	0.0140	−0.3351	3.6687	−1.2016	−0.0109		0.1123	3.4866	−1.2016	−0.0109	0.1123
	**w_9_**	**b_33_**	**w_30″_**	**w_31″_**	**w_32″_**	**b_4_**		**w_10_**	**w_11_**	**w_12_**	**w_12″_**	**b_5_**
DNN	3.4866	−12.5063	0.0677	−0.1792	11.4919	−4.8126		0.4069	0.8339	0.8339	6.1408	−4.8126
	**w_13_**	**w_14_**	**w_15_**	**w_15″_**	**b_6_**	**w_16_**	**w_17_**	**w_18_**	**w_18″_**	**b_66_**	**w_60″_**	**w_61″_**
DNN	0.4069	0.8339	0.8339	6.1408	−4.1466	1.2712	1.5218	1.5218	2.0836	3.5725	5.3155	4.5526
	**w_62″_**	**w_63″_**	**b_7_**	**w_19_**	**w_20_**	**w_21_**	**w_21″_**	**L_R_**	$\sum_{i=1}^{n}C$	$I_{N}$	$\varepsilon_{a}$	$\varepsilon_{t}$
DNN5	4.5526	2.2474	−8.9038	6.6057	6.6057	5.9931	7.0674	2.0	0.025	1,090,594	24.186	0.023

NB: b_i_ is the bias, w_i_ is the synaptic weight, L_R_ is the linearity rate, C is the cost function, $I_{N}$ is the number of iterations or loops, $\varepsilon_{a}$ is the approximate error and $\varepsilon_{t}$ is the true error. Training time (t) = 60 h~4 days.

## Data Availability

Data are contained within the article and Supplementary Materials.

## Supplementary Materials

The following supporting information can be downloaded at: https://www.mdpi.com/article/10.3390/polym16111515/s1. DNN frameworks and learning algorithms developed for this study are presented in this Supplementary Material.
