# Supplementary material for: Machine Learning Analysis of Enhanced Biodegradable Phoenix dactylifera L./HDPE Composite Thermograms
Source: Polymers (Basel). 2024 May 27;16(11):1515. doi: 10.3390/polym16111515 (PMC11174886; doi:10.3390/polym16111515)
Supplement: Supplementary file 1 [file polymers-16-01515-s001.zip › polymers-2984738-supplementary.pdf]

Supplementary Data

Learning Algorithms Developed for DNN framework in Figure 3

The analysis of general artificial neural networks (ANN) for determining the sum weight for the framework Figure 3 in the main text is expressed as:

$$z_j = \sum_i w_i . x_i + b_k \qquad \text{Eqn 1.0a}$$

The Sigmoid activation function (a) is defined as the logistic function of this sum weight ($\sigma'[z_j]$)

$$a_j = \sigma'[z_j] = \frac{1}{1+e^{-z_j}} \qquad \text{Eqn 1.0b}$$

The cost function is defined as the square of the difference between the real (y) and predicted (activation function, $[a_j]$) output signals.

$$C = (y - a_j)^2 \qquad \text{Eqn 1.0c}$$

Thus, applying Eqn 1a to the DNN framework in Fig 3 results in

$$z_7 = b_7 + w_{19}.a_4 + w_{20}.a_5 + w_{21}.a_6 \qquad \text{Eqn 1.1a}$$

$$a_7 = \sigma'(z_7) = \frac{1}{(1+e^{-z_7})} \qquad \text{Eqn 1.1b}$$

$$C = (y - a_7)^2 \qquad \text{Eqn 1.1c}$$

where $a_4 = \sigma'(z_4) = \frac{1}{(1+e^{-z_4})}$ & $z_4 = b_4 + w_{10}.a_1 + w_{11}.a_2 + w_{12}.a_3$ Eqn 1.2

$$a_5 = \sigma'(z_5) = \frac{1}{(1+e^{-z_5})} \ \& \ z_5 = b_5 + w_{13}.a_1 + w_{14}.a_2 + w_{15}.a_3 \qquad \text{Eqn 1.3}$$

$$a_6 = \sigma'(z_6) = \frac{1}{(1+e^{-z_6})} \ \& \ z_6 = b_6 + w_{16}.a_1 + w_{17}.a_2 + w_{18}.a_3 \qquad \text{Eqn 1.4}$$

$$a_1 = \sigma'(z_1) = \frac{1}{(1+e^{-z_1})} \ \& \ z_1 = b_1 + w_1.x_1 + w_2.x_2 + w_3.x_3 \qquad \text{Eqn 1.5}$$

$$a_2 = \sigma'(z_2) = \frac{1}{(1+e^{-z_2})} \ \& \ z_2 = b_2 + w_4.x_1 + w_5.x_2 + w_6.x_3 \qquad \text{Eqn 16}$$

$$a_3 = \sigma'(z_3) = \frac{1}{(1+e^{-z_3})} \ \& \ z_3 = b_3 + w_7.x_1 + w_8.x_2 + w_9.x_3 \qquad \text{Eqn 1.7}$$

where $b_1 - b_7$ & $w_1 - w_{21}$ are chosen arbitrarily constants, and their values can be determined through a process of trial and error. The goal is to find numerical values that closely match predictions (output 7, a_7) to experiments (y) as the overall cost function ($\sum_i C \sim 0$) should ideally reduce to zero. Though this trial-and-error approach may seem tedious and time-consuming, there is a possible way to address this issue through the application of the cost optimization technique. This technique involves optimizing the input signal of the DNN framework based on the output signal, as shown below.

Stage 1: The overall cost function on output 7

Starting with single-layer neural networks (SNN) from output 7, the analysis of the backpropagation network for the cost optimization approach begins with mathematical relations for the changes associated with the sum weight (Δw) and bias (Δb). These changes are expressed as a test function of linearity and their respective cost gradients, as shown in Eqn 2.0.

$$\Delta w = -R_L \frac{\partial C}{\partial w} \text{ and } \Delta b = -R_L \frac{\partial C}{\partial b} \qquad \text{Eqn 2.0}$$

where R_L is the learning rate which represent an arbitrary chosen value for the training of the dataset and

$$\frac{\partial C}{\partial w} = \left(\frac{\partial C}{\partial a}\right)\left(\frac{\partial a}{\partial z}\right)\left(\frac{\partial z}{\partial w}\right); \quad \frac{\partial C}{\partial b} = \left(\frac{\partial C}{\partial a}\right)\left(\frac{\partial a}{\partial z}\right)\left(\frac{\partial z}{\partial b}\right) \qquad \text{Eqn 2.1}$$

$$\left(\frac{\partial C}{\partial a}\right) = 2(a - y); \left(\frac{\partial a}{\partial z}\right) = \sigma'(z) = \frac{e^z}{(1+e^z)^2}; \left(\frac{\partial z}{\partial w}\right) = x; \quad \left(\frac{\partial z}{\partial b}\right) = 1 \qquad \text{Eqn 2.2}$$

The following equations are obtained by applying Eqns 2.1 and 2.2 of the cost minimizations to the backpropagation framework in Fig 3.

$$\left(\frac{\partial C}{\partial a_7}\right) = 2(a_7 - y); \quad \left(\frac{\partial a_7}{\partial z_7}\right) = \sigma'(z_7) = \frac{e^{z_7}}{(1+e^{z_7})^2}; \quad \left(\frac{\partial z_7}{\partial w_{19}}\right) = a_4, \left(\frac{\partial z_7}{\partial w_{20}}\right) = a_5, \left(\frac{\partial z_7}{\partial w_{21}}\right) = a_6,$$

$$\text{and } \left(\frac{\partial z_7}{\partial b_7}\right) = 1. \qquad \text{Eqn 2.3}$$

$$\left(\frac{\partial C}{\partial w_{19}}\right) = \left(\frac{\partial C}{\partial a_7}\right)\left(\frac{\partial a_7}{\partial z_7}\right)\left(\frac{\partial z_7}{\partial w_{19}}\right) \qquad \text{Eqn 2.4a}$$

$$\left(\frac{\partial C}{\partial w_{20}}\right) = \left(\frac{\partial C}{\partial a_7}\right)\left(\frac{\partial a_7}{\partial z_7}\right)\left(\frac{\partial z_7}{\partial w_{20}}\right) \qquad \text{Eqn 2.4b}$$

$$\left(\frac{\partial C}{\partial w_{21}}\right) = \left(\frac{\partial C}{\partial a_7}\right)\left(\frac{\partial a_7}{\partial z_7}\right)\left(\frac{\partial z_7}{\partial w_{21}}\right) \qquad \text{Eqn 2.4c}$$

$$\left(\frac{\partial C}{\partial b_7}\right) = \left(\frac{\partial C}{\partial a_7}\right)\left(\frac{\partial a_7}{\partial z_7}\right)\left(\frac{\partial z_7}{\partial b_7}\right) \qquad \text{Eqn 2.4d}$$

Hence: $\Delta w_{19} = -R_L\left(\sum_{i=1}^{n}\left(\frac{\partial C}{\partial w_{19}}\right)\right)/n \quad \& \; w_{19(New)} = w_{19(Old)} + \Delta w_{19}$ Eqn 2.4e

$$\Delta w_{20} = -R_L\left(\sum_{i=1}^{n}\left(\frac{\partial C}{\partial w_{20}}\right)\right)/n \quad \& \; w_{20(New)} = w_{20(Old)} + \Delta w_{20} \qquad \text{Eqn 2.4f}$$

$$\Delta w_{21} = -R_L\left(\sum_{i=1}^{n}\left(\frac{\partial C}{\partial w_{21}}\right)\right)/n \quad \& \; w_{21(New)} = w_{21(Old)} + \Delta w_{21} \qquad \text{Eqn 2.4g}$$

$$\Delta b_7 = -R_L\left(\sum_{i=1}^{n}\left(\frac{\partial C}{\partial b_7}\right)\right)/n \quad \& \; b_7 = b_7 + \Delta b_5 \qquad \text{Eqn 2.4h}$$

Stage 2: The overall cost function on hidden layer 2

The computation of changes in bias and weights at this stage of backpropagation requires the dependence of the overall cost function $(\partial C/\partial a_h)$ and the sum weight $(\partial z/\partial a_h)$ on the activation of the hidden neurons. These cost functions are expressed as follows:

$$\left(\frac{\partial z}{\partial a_h}\right) = w \quad \& \quad \left(\frac{\partial C}{\partial a_h}\right) = \left(\frac{\partial C}{\partial a_o}\right)\left(\frac{\partial a_o}{\partial z}\right)\left(\frac{\partial z}{\partial a_h}\right) \qquad \text{Eqn 3.0}$$

Thus, by applying Eqn 3.0 to the hidden neurons (4, 5 & 6) in the DNN framework (Fig 3), the following mathematical expressions are obtained

$$\left(\frac{\partial z_7}{\partial a_4}\right) = w_{19} \qquad \text{Eqn 3.1a}$$

$$\left(\frac{\partial z_7}{\partial a_5}\right) = w_{20} \qquad \text{Eqn 3.1b}$$

$$\left(\frac{\partial z_7}{\partial a_6}\right) = w_{21} \qquad \text{Eqn 3.1c}$$

$$\left(\frac{\partial C}{\partial a_4}\right) = \left(\frac{\partial C}{\partial a_7}\right)\left(\frac{\partial a_7}{\partial z_7}\right)\left(\frac{\partial z_7}{\partial a_4}\right) \quad \text{where} \quad \left(\frac{\partial a_7}{\partial z_7}\right) = \sigma'(z_7) = \frac{e^{z_7}}{(1+e^{z_7})^2} \qquad \text{Eqn 3.2a}$$

$$\left(\frac{\partial C}{\partial a_5}\right) = \left(\frac{\partial C}{\partial a_7}\right)\left(\frac{\partial a_7}{\partial z_7}\right)\left(\frac{\partial z_7}{\partial a_5}\right) \qquad \text{Eqn 3.2b}$$

$$\left(\frac{\partial C}{\partial a_6}\right) = \left(\frac{\partial C}{\partial a_7}\right)\left(\frac{\partial a_7}{\partial z_7}\right)\left(\frac{\partial z_7}{\partial a_6}\right) \qquad \text{Eqn 3.2c}$$

$$\left(\frac{\partial C}{\partial w_{10}}\right) = \left(\frac{\partial C}{\partial a_4}\right)\left(\frac{\partial a_4}{\partial z_4}\right)\left(\frac{\partial z_4}{\partial w_{10}}\right) \quad \text{where} \left(\frac{\partial z_4}{\partial w_{10}}\right) = a_1 \ \& \left(\frac{\partial a_4}{\partial z_4}\right) = \sigma'(z_4) = \frac{e^{z_4}}{(1+e^{z_4})^2} \qquad \text{Eqn 3.3a}$$

$$\left(\frac{\partial C}{\partial w_{11}}\right) = \left(\frac{\partial C}{\partial a_4}\right)\left(\frac{\partial a_4}{\partial z_4}\right)\left(\frac{\partial z_4}{\partial w_{11}}\right) \quad \text{where} \left(\frac{\partial z_4}{\partial w_{11}}\right) = a_2 \qquad \text{Eqn 3.3b}$$

$$\left(\frac{\partial C}{\partial w_{12}}\right) = \left(\frac{\partial C}{\partial a_4}\right)\left(\frac{\partial a_4}{\partial z_4}\right)\left(\frac{\partial z_4}{\partial w_{12}}\right) \quad \text{where} \left(\frac{\partial z_4}{\partial w_{12}}\right) = a_3 \qquad \text{Eqn 3.3c}$$

$$\left(\frac{\partial C}{\partial b_4}\right) = \left(\frac{\partial C}{\partial a_4}\right)\left(\frac{\partial a_4}{\partial z_4}\right)\left(\frac{\partial z_4}{\partial b_4}\right) \quad \text{where} \left(\frac{\partial z_4}{\partial b_4}\right) = 1 \qquad \text{Eqn 3.3d}$$

Hence: $\Delta w_{10} = -R_L\left(\sum_{i=1}^{n}\left(\frac{\partial C}{\partial w_{10}}\right)\right)/n \quad \& \ w_{10(New)} = w_{10(Old)} + \Delta w_{10}$ Eqn 3.3e

$$\Delta w_{11} = -R_L\left(\sum_{i=1}^{n}\left(\frac{\partial C}{\partial w_{11}}\right)\right)/n \quad \& \ w_{11(New)} = w_{11(Old)} + \Delta w_{11} \qquad \text{Eqn 3.3f}$$

$$\Delta w_{12} = -R_L\left(\sum_{i=1}^{n}\left(\frac{\partial C}{\partial w_{12}}\right)\right)/n \quad \& \ w_{12(New)} = w_{12(Old)} + \Delta w_{12} \qquad \text{Eqn 3.3g}$$

$$\Delta b_4 = -R_L\left(\sum_{i=1}^{n}\left(\frac{\partial C}{\partial b_4}\right)\right)/n \quad \& \ b_{4(New)} = b_{4(Old)} + \Delta b_4 \qquad \text{Eqn 3.3h}$$

$\left(\frac{\partial C}{\partial w_{13}}\right) = \left(\frac{\partial C}{\partial a_5}\right)\left(\frac{\partial a_5}{\partial z_5}\right)\left(\frac{\partial z_5}{\partial w_{13}}\right)$ where $\left(\frac{\partial z_5}{\partial w_{13}}\right) = a_1$ & $\left(\frac{\partial a_5}{\partial z_5}\right) = \sigma'(z_5) = \frac{e^{z_5}}{(1+e^{z_5})^2}$ Eqn 3.4a

$\left(\frac{\partial C}{\partial w_{14}}\right) = \left(\frac{\partial C}{\partial a_5}\right)\left(\frac{\partial a_5}{\partial z_5}\right)\left(\frac{\partial z_5}{\partial w_{14}}\right)$ where $\left(\frac{\partial z_5}{\partial w_{14}}\right) = a_2$ Eqn 3.4b

$\left(\frac{\partial C}{\partial w_{15}}\right) = \left(\frac{\partial C}{\partial a_5}\right)\left(\frac{\partial a_5}{\partial z_5}\right)\left(\frac{\partial z_5}{\partial w_{15}}\right)$ where $\left(\frac{\partial z_5}{\partial w_{15}}\right) = a_3$ Eqn 3.4c

$\left(\frac{\partial C}{\partial b_5}\right) = \left(\frac{\partial C}{\partial a_5}\right)\left(\frac{\partial a_5}{\partial z_5}\right)\left(\frac{\partial z_5}{\partial b_5}\right)$ where $\left(\frac{\partial z_5}{\partial b_5}\right) = 1$ Eqn 3.4d

Hence: $\Delta w_{13} = -R_L\left(\sum_{i=1}^{n}\left(\frac{\partial C}{\partial w_{13}}\right)\right)/n$ & $w_{13(New)} = w_{13(Old)} + \Delta w_{13}$ Eqn 3.4e

$\Delta w_{14} = -R_L\left(\sum_{i=1}^{n}\left(\frac{\partial C}{\partial w_{14}}\right)\right)/n$ & $w_{14(New)} = w_{14(Old)} + \Delta w_{14}$ Eqn 3.4f

$\Delta w_{15} = -R_L\left(\sum_{i=1}^{n}\left(\frac{\partial C}{\partial w_{15}}\right)\right)/n$ & $w_{15(New)} = w_{15(Old)} + \Delta w_{15}$ Eqn 3.4g

$\Delta b_5 = -R_L\left(\sum_{i=1}^{n}\left(\frac{\partial C}{\partial b_5}\right)\right)/n$ & $b_{5(New)} = b_{5(Old)} + \Delta b_5$ Eqn 3.4h

$\left(\frac{\partial C}{\partial w_{16}}\right) = \left(\frac{\partial C}{\partial a_6}\right)\left(\frac{\partial a_6}{\partial z_6}\right)\left(\frac{\partial z_6}{\partial w_{16}}\right)$ where $\left(\frac{\partial z_6}{\partial w_{16}}\right) = a_1$ & $\left(\frac{\partial a_6}{\partial z_6}\right) = \sigma'(z_6) = \frac{e^{z_6}}{(1+e^{z_6})^2}$ Eqn 3.5a

$\left(\frac{\partial C}{\partial w_{17}}\right) = \left(\frac{\partial C}{\partial a_6}\right)\left(\frac{\partial a_6}{\partial z_6}\right)\left(\frac{\partial z_6}{\partial w_{17}}\right)$ where $\left(\frac{\partial z_6}{\partial w_{17}}\right) = a_2$ Eqn 3.5b

$\left(\frac{\partial C}{\partial w_{18}}\right) = \left(\frac{\partial C}{\partial a_6}\right)\left(\frac{\partial a_6}{\partial z_6}\right)\left(\frac{\partial z_6}{\partial w_{18}}\right)$ where $\left(\frac{\partial z_6}{\partial w_{18}}\right) = a_3$ Eqn 3.5c

$\left(\frac{\partial C}{\partial b_6}\right) = \left(\frac{\partial C}{\partial a_6}\right)\left(\frac{\partial a_6}{\partial z_6}\right)\left(\frac{\partial z_6}{\partial b_6}\right)$ where $\left(\frac{\partial z_6}{\partial b_6}\right) = 1$ Eqn 3.5d

Hence: $\Delta w_{16} = -R_L\left(\sum_{i=1}^{n}\left(\frac{\partial C}{\partial w_{16}}\right)\right)/n$ & $w_{16(New)} = w_{16(Old)} + \Delta w_{16}$ Eqn 3.5e

$\Delta w_{17} = -R_L\left(\sum_{i=1}^{n}\left(\frac{\partial C}{\partial w_{17}}\right)\right)/n$ & $w_{17(New)} = w_{17(Old)} + \Delta w_{17}$ Eqn 3.5f

$\Delta w_{18} = -R_L\left(\sum_{i=1}^{n}\left(\frac{\partial C}{\partial w_{18}}\right)\right)/n$ & $w_{18(New)} = w_{18(Old)} + \Delta w_{18}$ Eqn 3.4g

$\Delta b_6 = -R_L\left(\sum_{i=1}^{n}\left(\frac{\partial C}{\partial b_6}\right)\right)/n$ & $b_{6(New)} = b_{6(Old)} + \Delta b_6$ Eqn 3.4h

Stage 3: The overall cost function on hidden layer 1

$\left(\frac{\partial C}{\partial a_1}\right)_A = \left(\frac{\partial C}{\partial a_4}\right)\left(\frac{\partial a_4}{\partial z_4}\right)\left(\frac{\partial z_4}{\partial a_1}\right)$ where $\left(\frac{\partial a_4}{\partial z_4}\right) = \sigma'(z_4) = \frac{e^{z_4}}{(1+e^{z_4})^2}$ & $\left(\frac{\partial z_4}{\partial a_1}\right) = w_{10}$ Eqn 4.0a

$$\left(\frac{\partial C}{\partial a_1}\right)_B = \left(\frac{\partial C}{\partial a_5}\right)\left(\frac{\partial a_5}{\partial z_5}\right)\left(\frac{\partial z_5}{\partial a_1}\right) \text{ where } \left(\frac{\partial a_5}{\partial z_5}\right) = \sigma'(z_5) = \frac{e^{z_5}}{(1+e^{z_5})^2} \ \& \ \left(\frac{\partial z_5}{\partial a_1}\right) = w_{13} \qquad \text{Eqn 4.0b}$$

$$\left(\frac{\partial C}{\partial a_1}\right)_C = \left(\frac{\partial C}{\partial a_6}\right)\left(\frac{\partial a_6}{\partial z_6}\right)\left(\frac{\partial z_6}{\partial a_1}\right) \text{ where } \left(\frac{\partial a_6}{\partial z_6}\right) = \sigma'(z_6) = \frac{e^{z_6}}{(1+e^{z_6})^2} \ \& \ \left(\frac{\partial z_6}{\partial a_1}\right) = w_{16} \qquad \text{Eqn 4.0c}$$

$$\left(\frac{\partial C}{\partial a_1}\right) = \left(\frac{\partial C}{\partial a_1}\right)_A + \left(\frac{\partial C}{\partial a_1}\right)_B + \left(\frac{\partial C}{\partial a_1}\right)_C \qquad \text{Eqn 4.0d}$$

$$\left(\frac{\partial C}{\partial a_2}\right)_A = \left(\frac{\partial C}{\partial a_4}\right)\left(\frac{\partial a_4}{\partial z_4}\right)\left(\frac{\partial z_4}{\partial a_2}\right) \text{ where } \left(\frac{\partial a_4}{\partial z_4}\right) = \sigma'(z_4) = \frac{e^{z_4}}{(1+e^{z_4})^2} \ \& \ \left(\frac{\partial z_4}{\partial a_2}\right) = w_{11} \qquad \text{Eqn 4.1a}$$

$$\left(\frac{\partial C}{\partial a_2}\right)_B = \left(\frac{\partial C}{\partial a_5}\right)\left(\frac{\partial a_5}{\partial z_5}\right)\left(\frac{\partial z_5}{\partial a_2}\right) \text{ where } \left(\frac{\partial a_5}{\partial z_5}\right) = \sigma'(z_5) = \frac{e^{z_5}}{(1+e^{z_5})^2} \ \& \ \left(\frac{\partial z_5}{\partial a_2}\right) = w_{14} \qquad \text{Eqn 4.1b}$$

$$\left(\frac{\partial C}{\partial a_2}\right)_C = \left(\frac{\partial C}{\partial a_6}\right)\left(\frac{\partial a_6}{\partial z_6}\right)\left(\frac{\partial z_6}{\partial a_2}\right) \text{ where } \left(\frac{\partial a_6}{\partial z_6}\right) = \sigma'(z_6) = \frac{e^{z_6}}{(1+e^{z_6})^2} \ \& \ \left(\frac{\partial z_6}{\partial a_2}\right) = w_{17} \qquad \text{Eqn 4.1c}$$

$$\left(\frac{\partial C}{\partial a_2}\right) = \left(\frac{\partial C}{\partial a_2}\right)_A + \left(\frac{\partial C}{\partial a_2}\right)_B + \left(\frac{\partial C}{\partial a_2}\right)_C \qquad \text{Eqn 4.1d}$$

$$\left(\frac{\partial C}{\partial a_3}\right)_A = \left(\frac{\partial C}{\partial a_4}\right)\left(\frac{\partial a_4}{\partial z_4}\right)\left(\frac{\partial z_4}{\partial a_3}\right) \text{ where } \left(\frac{\partial a_4}{\partial z_4}\right) = \sigma'(z_4) = \frac{e^{z_4}}{(1+e^{z_4})^2} \ \& \ \left(\frac{\partial z_4}{\partial a_3}\right) = w_{12} \qquad \text{Eqn 4.2a}$$

$$\left(\frac{\partial C}{\partial a_3}\right)_B = \left(\frac{\partial C}{\partial a_5}\right)\left(\frac{\partial a_5}{\partial z_5}\right)\left(\frac{\partial z_5}{\partial a_3}\right) \text{ where } \left(\frac{\partial a_5}{\partial z_5}\right) = \sigma'(z_5) = \frac{e^{z_5}}{(1+e^{z_5})^2} \ \& \ \left(\frac{\partial z_5}{\partial a_3}\right) = w_{15} \qquad \text{Eqn 4.2b}$$

$$\left(\frac{\partial C}{\partial a_3}\right)_C = \left(\frac{\partial C}{\partial a_6}\right)\left(\frac{\partial a_6}{\partial z_6}\right)\left(\frac{\partial z_6}{\partial a_3}\right) \text{ where } \left(\frac{\partial a_6}{\partial z_6}\right) = \sigma'(z_6) = \frac{e^{z_6}}{(1+e^{z_6})^2} \ \& \ \left(\frac{\partial z_6}{\partial a_3}\right) = w_{18} \qquad \text{Eqn 4.2c}$$

$$\left(\frac{\partial C}{\partial a_3}\right) = \left(\frac{\partial C}{\partial a_3}\right)_A + \left(\frac{\partial C}{\partial a_3}\right)_B + \left(\frac{\partial C}{\partial a_3}\right)_C \qquad \text{Eqn 4.2d}$$

Stage 4: The overall cost function on inputs 1, 2 and 3

This approach requires going back to the familiar territory of adopting single-layer networks (SNN) to formulate the propagation between the inputs and hidden neurons 1. This entails applying Eqns 2.0 – 2.2 to compute the changes in the cost function associated with changes in synaptic weights and biases, to achieve the following mathematical expressions:

$$\left(\frac{\partial C}{\partial w_1}\right) = \left(\frac{\partial C}{\partial a_1}\right)\left(\frac{\partial a_1}{\partial z_1}\right)\left(\frac{\partial z_1}{\partial w_1}\right) \text{ where } \left(\frac{\partial z_1}{\partial w_1}\right) = x_1 \ \& \ \left(\frac{\partial a_1}{\partial z_1}\right) = \sigma'(z_1) = \frac{e^{z_1}}{(1+e^{z_1})^2} \qquad \text{Eqn 5.0a}$$

$$\left(\frac{\partial C}{\partial w_2}\right) = \left(\frac{\partial C}{\partial a_1}\right)\left(\frac{\partial a_1}{\partial z_1}\right)\left(\frac{\partial z_1}{\partial w_2}\right) \text{ where } \left(\frac{\partial z_1}{\partial w_2}\right) = x_2 \qquad \text{Eqn 5.0b}$$

$$\left(\frac{\partial C}{\partial w_3}\right) = \left(\frac{\partial C}{\partial a_1}\right)\left(\frac{\partial a_1}{\partial z_1}\right)\left(\frac{\partial z_1}{\partial w_3}\right) \text{ where } \left(\frac{\partial z_1}{\partial w_3}\right) = x_3 \qquad \text{Eqn 5.0c}$$

$$\left(\frac{\partial C}{\partial b_1}\right) = \left(\frac{\partial C}{\partial a_1}\right)\left(\frac{\partial a_1}{\partial z_1}\right)\left(\frac{\partial z_1}{\partial b_1}\right) \text{ where } \left(\frac{\partial z_1}{\partial b_1}\right) = 1 \qquad \text{Eqn 5.0d}$$

Hence: $\Delta w_1 = -R_L\left(\sum_{i=1}^{n}\left(\frac{\partial C}{\partial w_1}\right)\right)/n$ & $w_{1(New)} = w_{1(Old)} + \Delta w_1$ Eqn 5.1a

$\Delta w_2 = -R_L\left(\sum_{i=1}^{n}\left(\frac{\partial C}{\partial w_2}\right)\right)/n$ & $w_{2(New)} = w_{2(Old)} + \Delta w_2$ Eqn 5.1b

$\Delta w_3 = -R_L\left(\sum_{i=1}^{n}\left(\frac{\partial C}{\partial w_3}\right)\right)/n$ & $w_{3(New)} = w_{3(Old)} + \Delta w_3$ Eqn 5.1c

$\Delta b_1 = -R_L\left(\sum_{i=1}^{n}\left(\frac{\partial C}{\partial b_1}\right)\right)/n$ & $b_{1(New)} = b_{1(Old)} + \Delta b_1$ Eqn 5.1d

$$\left(\frac{\partial C}{\partial w_4}\right) = \left(\frac{\partial C}{\partial a_2}\right)\left(\frac{\partial a_2}{\partial z_2}\right)\left(\frac{\partial z_2}{\partial w_4}\right) \text{ where } \left(\frac{\partial z_2}{\partial w_4}\right) = x_1 \text{ \& } \left(\frac{\partial a_2}{\partial z_2}\right) = \sigma'(z_2) = \frac{e^{z_2}}{(1+e^{z_2})^2} \qquad \text{Eqn 5.2a}$$

$$\left(\frac{\partial C}{\partial w_5}\right) = \left(\frac{\partial C}{\partial a_2}\right)\left(\frac{\partial a_2}{\partial z_2}\right)\left(\frac{\partial z_2}{\partial w_5}\right) \text{ where } \left(\frac{\partial z_2}{\partial w_5}\right) = x_2 \qquad \text{Eqn 5.2b}$$

$$\left(\frac{\partial C}{\partial w_6}\right) = \left(\frac{\partial C}{\partial a_2}\right)\left(\frac{\partial a_2}{\partial z_2}\right)\left(\frac{\partial z_2}{\partial w_6}\right) \text{ where } \left(\frac{\partial z_2}{\partial w_6}\right) = x_3 \qquad \text{Eqn 5.2c}$$

$$\left(\frac{\partial C}{\partial b_2}\right) = \left(\frac{\partial C}{\partial a_2}\right)\left(\frac{\partial a_2}{\partial z_2}\right)\left(\frac{\partial z_2}{\partial b_2}\right) \text{ where } \left(\frac{\partial z_2}{\partial b_2}\right) = 1 \qquad \text{Eqn 5.2d}$$

Hence: $\Delta w_4 = -R_L\left(\sum_{i=1}^{n}\left(\frac{\partial C}{\partial w_4}\right)\right)/n$ & $w_{4(New)} = w_{4(Old)} + \Delta w_4$ Eqn 5.3a

$\Delta w_5 = -R_L\left(\sum_{i=1}^{n}\left(\frac{\partial C}{\partial w_5}\right)\right)/n$ & $w_{5(New)} = w_{5(Old)} + \Delta w_5$ Eqn 5.3b

$\Delta w_6 = -R_L\left(\sum_{i=1}^{n}\left(\frac{\partial C}{\partial w_6}\right)\right)/n$ & $w_{6(New)} = w_{6(Old)} + \Delta w_6$ Eqn 5.3c

$\Delta b_2 = -R_L\left(\sum_{i=1}^{n}\left(\frac{\partial C}{\partial b_2}\right)\right)/n$ & $b_{2(New)} = b_{2(Old)} + \Delta b_2$ Eqn 5.3d

$$\left(\frac{\partial C}{\partial w_7}\right) = \left(\frac{\partial C}{\partial a_3}\right)\left(\frac{\partial a_3}{\partial z_3}\right)\left(\frac{\partial z_3}{\partial w_7}\right) \text{ where } \left(\frac{\partial z_3}{\partial w_7}\right) = x_1 \text{ \& } \left(\frac{\partial a_3}{\partial z_3}\right) = \sigma'(z_3) = \frac{e^{z_3}}{(1+e^{z_3})^2} \qquad \text{Eqn 5.4a}$$

$$\left(\frac{\partial C}{\partial w_8}\right) = \left(\frac{\partial C}{\partial a_3}\right)\left(\frac{\partial a_3}{\partial z_3}\right)\left(\frac{\partial z_3}{\partial w_8}\right) \text{ where } \left(\frac{\partial z_3}{\partial w_8}\right) = x_2 \qquad \text{Eqn 5.4b}$$

$$\left(\frac{\partial C}{\partial w_9}\right) = \left(\frac{\partial C}{\partial a_3}\right)\left(\frac{\partial a_3}{\partial z_3}\right)\left(\frac{\partial z_3}{\partial w_9}\right) \text{ where } \left(\frac{\partial z_3}{\partial w_9}\right) = x_3 \qquad \text{Eqn 5.4c}$$

$$\left(\frac{\partial C}{\partial b_3}\right) = \left(\frac{\partial C}{\partial a_3}\right)\left(\frac{\partial a_3}{\partial z_3}\right)\left(\frac{\partial z_3}{\partial b_3}\right) \text{ where } \left(\frac{\partial z_3}{\partial b_3}\right) = 1 \qquad \text{Eqn 5.4d}$$

Hence: $\Delta w_7 = -R_L\left(\sum_{i=1}^{n}\left(\frac{\partial C}{\partial w_7}\right)\right)/n$ & $w_{7(New)} = w_{7(Old)} + \Delta w_7$ Eqn 5.5a

$\Delta w_8 = -R_L\left(\sum_{i=1}^{n}\left(\frac{\partial C}{\partial w_8}\right)\right)/n$ & $w_{8(New)} = w_{8(Old)} + \Delta w_8$ Eqn 5.5b

$\Delta w_9 = -R_L\left(\sum_{i=1}^{n}\left(\frac{\partial C}{\partial w_9}\right)\right)/n$ & $w_{9(New)} = w_{9(Old)} + \Delta w_9$ Eqn 5.6c

$\Delta b_3 = -R_L\left(\sum_{i=1}^{n}\left(\frac{\partial C}{\partial b_3}\right)\right)/n$ & $b_{3(New)} = b_{3(Old)} + \Delta b_3$ Eqn 5.6d

Thus a summary of the formulated learning algorithms needed for the optimization of the training cost function, which represents the residual error or simply deviations between the experimental and predicted signals, is provided by equations 5.7 – 5.10.

Stage 1: The overall cost function on output 7

$$\left(\frac{\partial C}{\partial a_7}\right); \left(\frac{\partial a_7}{\partial z_7}\right); \left(\frac{\partial z_7}{\partial b_7}\right), \left(\frac{\partial z_7}{\partial w_{19}}\right), \left(\frac{\partial z_7}{\partial w_{20}}\right), \left(\frac{\partial z_7}{\partial w_{21}}\right); \left(\frac{\partial C}{\partial b_7}\right), \left(\frac{\partial C}{\partial w_{19}}\right), \left(\frac{\partial C}{\partial w_{20}}\right), \left(\frac{\partial C}{\partial w_{21}}\right) \quad 5.7$$

Stage 2: The overall cost function on hidden layer 2

$$\left.\begin{array}{l}
\left(\frac{\partial z_7}{\partial a_4}\right), \left(\frac{\partial z_7}{\partial a_5}\right), \left(\frac{\partial z_7}{\partial a_6}\right); \left(\frac{\partial C}{\partial a_4}\right), \left(\frac{\partial C}{\partial a_5}\right), \left(\frac{\partial C}{\partial a_6}\right); \\
\left(\frac{\partial z_4}{\partial b_4}\right), \left(\frac{\partial z_4}{\partial w_{10}}\right), \left(\frac{\partial z_4}{\partial w_{11}}\right), \left(\frac{\partial z_4}{\partial w_{12}}\right); \left(\frac{\partial a_4}{\partial z_4}\right); \left(\frac{\partial C}{\partial b_4}\right), \left(\frac{\partial C}{\partial w_{10}}\right), \left(\frac{\partial C}{\partial w_{11}}\right), \left(\frac{\partial C}{\partial w_{12}}\right); \\
\left(\frac{\partial z_5}{\partial b_5}\right), \left(\frac{\partial z_5}{\partial w_{13}}\right), \left(\frac{\partial z_5}{\partial w_{14}}\right), \left(\frac{\partial z_5}{\partial w_{15}}\right); \left(\frac{\partial a_5}{\partial z_5}\right); \left(\frac{\partial C}{\partial b_5}\right), \left(\frac{\partial C}{\partial w_{13}}\right), \left(\frac{\partial C}{\partial w_{14}}\right), \left(\frac{\partial C}{\partial w_{15}}\right); \\
\left(\frac{\partial z_6}{\partial b_6}\right), \left(\frac{\partial z_6}{\partial w_{16}}\right), \left(\frac{\partial z_6}{\partial w_{17}}\right), \left(\frac{\partial z_6}{\partial w_{18}}\right); \left(\frac{\partial a_6}{\partial z_6}\right); \left(\frac{\partial C}{\partial b_6}\right), \left(\frac{\partial C}{\partial w_{16}}\right), \left(\frac{\partial C}{\partial w_{17}}\right), \left(\frac{\partial C}{\partial w_{18}}\right);
\end{array}\right\} \quad 5.8$$

Stage 3: The overall cost function on hidden layer 1

$$\left.\begin{array}{l}
\left(\frac{\partial z_4}{\partial a_1}\right), \left(\frac{\partial z_5}{\partial a_1}\right), \left(\frac{\partial z_6}{\partial a_1}\right); \left(\frac{\partial C}{\partial a_1}\right)_A, \left(\frac{\partial C}{\partial a_1}\right)_B, \left(\frac{\partial C}{\partial a_1}\right)_C; \left(\frac{\partial C}{\partial a_1}\right); \\
\left(\frac{\partial z_4}{\partial a_2}\right), \left(\frac{\partial z_5}{\partial a_2}\right), \left(\frac{\partial z_6}{\partial a_2}\right); \left(\frac{\partial C}{\partial a_2}\right)_A, \left(\frac{\partial C}{\partial a_2}\right)_B, \left(\frac{\partial C}{\partial a_2}\right)_C; \left(\frac{\partial C}{\partial a_2}\right); \\
\left(\frac{\partial z_4}{\partial a_3}\right), \left(\frac{\partial z_5}{\partial a_2}\right), \left(\frac{\partial z_6}{\partial a_3}\right); \left(\frac{\partial C}{\partial a_3}\right)_A, \left(\frac{\partial C}{\partial a_3}\right)_B, \left(\frac{\partial C}{\partial a_3}\right)_C; \left(\frac{\partial C}{\partial a_3}\right);
\end{array}\right\} \quad 5.9$$

Stage 4: The overall cost function on inputs 1, 2 and 3

$$\left.\begin{array}{l}
\left(\frac{\partial z_1}{\partial b_1}\right), \left(\frac{\partial z_1}{\partial w_1}\right), \left(\frac{\partial z_1}{\partial w_2}\right), \left(\frac{\partial z_1}{\partial w_3}\right); \left(\frac{\partial a_1}{\partial z_1}\right), \left(\frac{\partial C}{\partial a_1}\right); \left(\frac{\partial C}{\partial b_1}\right), \left(\frac{\partial C}{\partial w_1}\right), \left(\frac{\partial C}{\partial w_2}\right), \left(\frac{\partial C}{\partial w_3}\right);
\end{array}\right\} \quad 5.10$$

$\left(\frac{\partial z_2}{\partial b_2}\right), \left(\frac{\partial z_2}{\partial w_4}\right), \left(\frac{\partial z_2}{\partial w_5}\right), \left(\frac{\partial z_2}{\partial w_6}\right); \left(\frac{\partial a_2}{\partial z_2}\right), \left(\frac{\partial C}{\partial a_2}\right); \left(\frac{\partial C}{\partial b_2}\right), \left(\frac{\partial C}{\partial w_4}\right), \left(\frac{\partial C}{\partial w_5}\right), \left(\frac{\partial C}{\partial w_6}\right);$

$\left(\frac{\partial z_3}{\partial b_3}\right), \left(\frac{\partial z_3}{\partial w_7}\right), \left(\frac{\partial z_3}{\partial w_8}\right), \left(\frac{\partial z_3}{\partial w_9}\right); \left(\frac{\partial a_3}{\partial z_3}\right), \left(\frac{\partial C}{\partial a_3}\right); \left(\frac{\partial C}{\partial b_3}\right), \left(\frac{\partial C}{\partial w_7}\right), \left(\frac{\partial C}{\partial w_8}\right), \left(\frac{\partial C}{\partial w_9}\right);$

Learning Algorithms Developed for DNN framework in Figure 5

Applying Eqns 1.0a and 1.0b to the framework in Fig 5 (main article) results in the following changes to the mathematical relations for the sum weights (z_i) and the activation function (a_i). The changes are presented as follows:

$z_7 = b_7 + w_{19}.a_4 + w_{20}.a_5 + w_{21}.a_6 + \boldsymbol{w''_{21} a_{66}}$ Eqn 6.0a

$a_7 = \sigma'(z_7) = \frac{1}{(1+e^{-z_7})}$ Eqn 6.0b

$C = (y - a_7)^2$ Eqn 6.0c

where $a_4 = \sigma'(z_4) = \frac{1}{(1+e^{-z_4})}$ & $z_4 = b_4 + w_{10}.a_1 + w_{11}.a_2 + w_{12}.a_3 + \boldsymbol{w''_{12} a_{33}}$ Eqn 6.1a

$a_5 = \sigma'(z_5) = \frac{1}{(1+e^{-z_5})}$ & $z_5 = b_5 + w_{13}.a_1 + w_{14}.a_2 + w_{15}.a_3 + \boldsymbol{w''_{15} a_{33}}$ Eqn 6.1b

$a_6 = \sigma'(z_6) = \frac{1}{(1+e^{-z_6})}$ & $z_6 = b_6 + w_{16}.a_1 + w_{17}.a_2 + w_{18}.a_3 + \boldsymbol{w''_{18} a_{33}}$ Eqn 6.1c

$\boldsymbol{a_{66} = \sigma'(z_{66}) = \frac{1}{(1+e^{-z_{66}})}}$ & $\boldsymbol{z_{66} = b_{66} + w''_{60} a_1 + w''_{61} a_2 + w''_{62} a_3 + w''_{63} a_{33}}$ Eqn 6.1d

$a_1 = \sigma'(z_1) = \frac{1}{(1+e^{-z_1})}$ & $z_1 = b_1 + w_1.x_1 + w_2.x_2 + w_3.x_3$ Eqn 6.1e

$a_2 = \sigma'(z_2) = \frac{1}{(1+e^{-z_2})}$ & $z_2 = b_2 + w_4.x_1 + w_5.x_2 + w_6.x_3$ Eqn 6.1f

$a_3 = \sigma'(z_3) = \frac{1}{(1+e^{-z_3})}$ & $z_3 = b_3 + w_7.x_1 + w_8.x_2 + w_9.x_3$ Eqn 6.1g

$\boldsymbol{a_{33} = \sigma'(z_{33}) = \frac{1}{(1+e^{-z_{33}})}}$ & $\boldsymbol{z_{33} = b_{33} + w''_{30} x_1 + w''_{31} x_2 + w''_{32} x_3}$ Eqn 6.1h

Stage 1: The overall cost function on output 7

The following modifications are made to Eqns 2.3 – 2.4h to address the cost optimization function relating to the output signal 7 in Fig 4.

$\left(\frac{\partial C}{\partial a_7}\right) = 2(a_7 - y); \left(\frac{\partial a_7}{\partial z_7}\right) = \sigma'(z_7) = \frac{e^{z_7}}{(1+e^{z_7})^2}; \left(\frac{\partial z_7}{\partial w_{19}}\right) = a_4, \left(\frac{\partial z_7}{\partial w_{20}}\right) = a_5, \left(\frac{\partial z_7}{\partial w_{21}}\right) = a_6, \left(\frac{\boldsymbol{\partial z_7}}{\boldsymbol{\partial w''_{21}}}\right) =$ $\boldsymbol{a_{66}}$ and $\left(\frac{\partial z_7}{\partial b_7}\right) = 1$ Eqn 6.2a

$\left(\frac{\boldsymbol{\partial C}}{\boldsymbol{\partial w''_{21}}}\right) = \left(\frac{\boldsymbol{\partial C}}{\boldsymbol{\partial a_7}}\right)\left(\frac{\boldsymbol{\partial a_7}}{\boldsymbol{\partial z_7}}\right)\left(\frac{\boldsymbol{\partial z_7}}{\boldsymbol{\partial w''_{21}}}\right)$ Eqn 6.2b

Hence: $\Delta \boldsymbol{w}''_{21} = -\boldsymbol{R_L}\left(\sum_{i=1}^{n}\left(\frac{\partial C}{\partial w''_{21}}\right)\right)/\boldsymbol{n}$ & $\boldsymbol{w}''_{21(New)} = \boldsymbol{w}''_{21(Old)} + \Delta \boldsymbol{w}''_{21}$ Eqn 6.3c

Stage 2: The overall cost function on hidden layer 2

The mathematical additions for Eqns 3.1a to 3.3h, which correspond to this modelling stage after implementing the new framework shown in Fig 4, are presented below.

$\left(\frac{\partial z_7}{\partial a_{66}}\right) = \boldsymbol{w}''_{21}$ Eqn 6.4a

$\left(\frac{\partial C}{\partial a_{66}}\right) = \left(\frac{\partial C}{\partial a_7}\right)\left(\frac{\partial a_7}{\partial z_7}\right)\left(\frac{\partial z_7}{\partial a_{66}}\right)$ where $\left(\frac{\partial a_7}{\partial z_7}\right) = \boldsymbol{\sigma}'(\boldsymbol{z_7}) = \frac{e^{z_7}}{(1+e^{z_7})^2}$ Eqn 6.4b

$\left(\frac{\partial C}{\partial w''_{12}}\right) = \left(\frac{\partial C}{\partial a_4}\right)\left(\frac{\partial a_4}{\partial z_4}\right)\left(\frac{\partial z_4}{\partial w''_{12}}\right)$ where $\left(\frac{\partial z_4}{\partial w''_{12}}\right) = \boldsymbol{a_{33}}$ & $\left(\frac{\partial a_4}{\partial z_4}\right) = \boldsymbol{\sigma}'(\boldsymbol{z_4}) = \frac{e^{z_4}}{(1+e^{z_4})^2}$ Eqn 6.4c

$\left(\frac{\partial C}{\partial w''_{15}}\right) = \left(\frac{\partial C}{\partial a_5}\right)\left(\frac{\partial a_5}{\partial z_5}\right)\left(\frac{\partial z_5}{\partial w''_{15}}\right)$ where $\left(\frac{\partial z_4}{\partial w''_{15}}\right) = \boldsymbol{a_{33}}$& $\left(\frac{\partial a_5}{\partial z_5}\right) = \boldsymbol{\sigma}'(\boldsymbol{z_5}) = \frac{e^{z_5}}{(1+e^{z_5})^2}$ Eqn 6.4d

$\left(\frac{\partial C}{\partial w''_{18}}\right) = \left(\frac{\partial C}{\partial a_6}\right)\left(\frac{\partial a_6}{\partial z_6}\right)\left(\frac{\partial z_6}{\partial w''_{18}}\right)$ where $\left(\frac{\partial z_6}{\partial w''_{18}}\right) = \boldsymbol{a_{33}}$ & $\left(\frac{\partial a_6}{\partial z_6}\right) = \boldsymbol{\sigma}'(\boldsymbol{z_6}) = \frac{e^{z_6}}{(1+e^{z_6})^2}$ Eqn 6.4e

Hence: $\Delta \boldsymbol{w}''_{12} = -R_L\left(\sum_{i=1}^{n}\left(\frac{\partial C}{\partial w''_{12}}\right)\right)/n$ & $\boldsymbol{w}''_{12(New)} = \boldsymbol{w}''_{12(Old)} + \Delta \boldsymbol{w}''_{12}$ Eqn 6.4f

$\Delta \boldsymbol{w}''_{15} = -R_L\left(\sum_{i=1}^{n}\left(\frac{\partial C}{\partial w''_{15}}\right)\right)/n$ & $\boldsymbol{w}''_{15(New)} = \boldsymbol{w}''_{15(Old)} + \Delta \boldsymbol{w}''_{15}$ Eqn 6.4g

$\Delta \boldsymbol{w}''_{18} = -R_L\left(\sum_{i=1}^{n}\left(\frac{\partial C}{\partial w''_{18}}\right)\right)/n$ & $\boldsymbol{w}''_{18(New)} = \boldsymbol{w}''_{18(Old)} + \Delta \boldsymbol{w}''_{18}$ Eqn 6.4h

$\left(\frac{\partial C}{\partial w''_{60}}\right) = \left(\frac{\partial C}{\partial a_{66}}\right)\left(\frac{\partial a_{66}}{\partial z_{66}}\right)\left(\frac{\partial z_{66}}{\partial w''_{60}}\right)$ where $\left(\frac{\partial z_{66}}{\partial w''_{60}}\right) = \boldsymbol{a_1}$ & $\left(\frac{\partial a_{66}}{\partial z_{66}}\right) = \boldsymbol{\sigma}'(\boldsymbol{z_{66}}) = \frac{e^{z_{66}}}{(1+e^{z_{66}})^2}$ Eqn 6.5a

$\left(\frac{\partial C}{\partial w''_{61}}\right) = \left(\frac{\partial C}{\partial a_{66}}\right)\left(\frac{\partial a_{66}}{\partial z_{66}}\right)\left(\frac{\partial z_{66}}{\partial w''_{61}}\right)$ where $\left(\frac{\partial z_{66}}{\partial w''_{61}}\right) = \boldsymbol{a_2}$ Eqn 6.5b

$\left(\frac{\partial C}{\partial w''_{62}}\right) = \left(\frac{\partial C}{\partial a_{66}}\right)\left(\frac{\partial a_{66}}{\partial z_{66}}\right)\left(\frac{\partial z_{66}}{\partial w''_{62}}\right)$ where $\left(\frac{\partial z_{66}}{\partial w''_{62}}\right) = \boldsymbol{a_3}$ Eqn 6.5c

$\left(\frac{\partial C}{\partial w''_{63}}\right) = \left(\frac{\partial C}{\partial a_{66}}\right)\left(\frac{\partial a_{66}}{\partial z_{66}}\right)\left(\frac{\partial z_{66}}{\partial w''_{63}}\right)$ where $\left(\frac{\partial z_{66}}{\partial w''_{63}}\right) = \boldsymbol{a_{33}}$ Eqn 6.5d

$\left(\frac{\partial C}{\partial b_{66}}\right) = \left(\frac{\partial C}{\partial a_{66}}\right)\left(\frac{\partial a_{66}}{\partial z_{66}}\right)\left(\frac{\partial z_{66}}{\partial b_{66}}\right)$ where $\left(\frac{\partial z_{66}}{\partial b_{66}}\right) = \boldsymbol{1}$ Eqn 6.5e

Hence: $\Delta \boldsymbol{w}''_{60} = -\boldsymbol{R_L}\left(\sum_{i=1}^{n}\left(\frac{\partial C}{\partial w''_{60}}\right)\right)/\boldsymbol{n}$ & $\boldsymbol{w}''_{60(New)} = \boldsymbol{w}''_{60(Old)} + \Delta \boldsymbol{w}''_{60}$ Eqn 6.6a

$\Delta \boldsymbol{w}''_{61} = -\boldsymbol{R_L}\left(\sum_{i=1}^{n}\left(\frac{\partial C}{\partial w''_{61}}\right)\right)/\boldsymbol{n}$ & $\boldsymbol{w}''_{61(New)} = \boldsymbol{w}''_{61(Old)} + \Delta \boldsymbol{w}''_{61}$ Eqn 6.6b

$\Delta \boldsymbol{w}''_{62} = -\boldsymbol{R_L}\left(\sum_{i=1}^{n}\left(\frac{\partial C}{\partial w''_{62}}\right)\right)/\boldsymbol{n}$ & $\boldsymbol{w}''_{62(New)} = \boldsymbol{w}''_{62(Old)} + \Delta \boldsymbol{w}''_{62}$ Eqn 6.6c

$$\Delta w_{63}'' = -R_L\left(\sum_{i=1}^{n}\left(\frac{\partial C}{\partial w_{63}''}\right)\right)/n \quad \& \; w_{63(New)}'' = w_{63(Old)}'' + \Delta w_{63}'' \qquad \text{Eqn 6.6d}$$

$$\Delta b_{66} = -R_L\left(\sum_{i=1}^{n}\left(\frac{\partial C}{\partial b_{66}}\right)\right)/n \quad \& \; b_{66(New)} = b_{66(Old)} + \Delta b_{66} \qquad \text{Eqn 6.6e}$$

Stage 3: The overall cost function on hidden layer 1

The new mathematical additions to Eqns 4.0a to 4.2d for this modelling stage, following the application of the new framework in Fig 4, are presented below.

$$\left(\frac{\partial C}{\partial a_1}\right)_D = \left(\frac{\partial C}{\partial a_{66}}\right)\left(\frac{\partial a_{66}}{\partial z_{66}}\right)\left(\frac{\partial z_{66}}{\partial a_1}\right) \text{ where } \left(\frac{\partial a_{66}}{\partial z_{66}}\right) = \sigma'(z_{66}) = \frac{e^{z_{66}}}{(1+e^{z_{66}})^2} \;\&\; \left(\frac{\partial z_{66}}{\partial a_1}\right) = w_{60}'' \qquad \text{Eqn 6.7a}$$

$$\left(\frac{\partial C}{\partial a_1}\right) = \left(\frac{\partial C}{\partial a_1}\right)_A + \left(\frac{\partial C}{\partial a_1}\right)_B + \left(\frac{\partial C}{\partial a_1}\right)_C + \left(\frac{\partial C}{\partial a_1}\right)_D \qquad \text{Eqn 6.7b}$$

$$\left(\frac{\partial C}{\partial a_2}\right)_D = \left(\frac{\partial C}{\partial a_{66}}\right)\left(\frac{\partial a_{66}}{\partial z_{66}}\right)\left(\frac{\partial z_{66}}{\partial a_2}\right) \text{ where } \left(\frac{\partial a_{66}}{\partial z_{66}}\right) = \sigma'(z_{66}) = \frac{e^{z_{66}}}{(1+e^{z_{66}})^2} \;\&\; \left(\frac{\partial z_{66}}{\partial a_2}\right) = w_{61}'' \qquad \text{Eqn 6.8a}$$

$$\left(\frac{\partial C}{\partial a_2}\right) = \left(\frac{\partial C}{\partial a_2}\right)_A + \left(\frac{\partial C}{\partial a_2}\right)_B + \left(\frac{\partial C}{\partial a_2}\right)_C + \left(\frac{\partial C}{\partial a_2}\right)_D \qquad \text{Eqn 6.8b}$$

$$\left(\frac{\partial C}{\partial a_3}\right)_D = \left(\frac{\partial C}{\partial a_{66}}\right)\left(\frac{\partial a_{66}}{\partial z_{66}}\right)\left(\frac{\partial z_{66}}{\partial a_3}\right) \text{ where } \left(\frac{\partial a_{66}}{\partial z_{66}}\right) = \sigma'(z_{66}) = \frac{e^{z_{66}}}{(1+e^{z_{66}})^2} \;\&\; \left(\frac{\partial z_{66}}{\partial a_3}\right) = w_{62}'' \qquad \text{Eqn 6.9a}$$

$$\left(\frac{\partial C}{\partial a_3}\right) = \left(\frac{\partial C}{\partial a_3}\right)_A + \left(\frac{\partial C}{\partial a_3}\right)_B + \left(\frac{\partial C}{\partial a_3}\right)_C + \left(\frac{\partial C}{\partial a_3}\right)_D \qquad \text{Eqn 6.9b}$$

$$\left(\frac{\partial C}{\partial a_{33}}\right)_A = \left(\frac{\partial C}{\partial a_4}\right)\left(\frac{\partial a_4}{\partial z_4}\right)\left(\frac{\partial z_4}{\partial a_{33}}\right) \text{ where } \left(\frac{\partial a_4}{\partial z_4}\right) = \sigma'(z_4) = \frac{e^{z_4}}{(1+e^{z_4})^2} \;\&\; \left(\frac{\partial z_4}{\partial a_{33}}\right) = w_{12}'' \qquad \text{Eqn 6.10a}$$

$$\left(\frac{\partial C}{\partial a_{33}}\right)_B = \left(\frac{\partial C}{\partial a_5}\right)\left(\frac{\partial a_5}{\partial z_5}\right)\left(\frac{\partial z_5}{\partial a_{33}}\right) \text{ where } \left(\frac{\partial a_5}{\partial z_5}\right) = \sigma'(z_5) = \frac{e^{z_5}}{(1+e^{z_5})^2} \;\&\; \left(\frac{\partial z_5}{\partial a_{33}}\right) = w_{15}'' \qquad \text{Eqn 6.10b}$$

$$\left(\frac{\partial C}{\partial a_{33}}\right)_C = \left(\frac{\partial C}{\partial a_6}\right)\left(\frac{\partial a_6}{\partial z_6}\right)\left(\frac{\partial z_6}{\partial a_{33}}\right) \text{ where } \left(\frac{\partial a_6}{\partial z_6}\right) = \sigma'(z_6) = \frac{e^{z_6}}{(1+e^{z_6})^2} \;\&\; \left(\frac{\partial z_6}{\partial a_{33}}\right) = w_{18}'' \qquad \text{Eqn 6.10c}$$

$$\left(\frac{\partial C}{\partial a_{33}}\right)_D = \left(\frac{\partial C}{\partial a_{66}}\right)\left(\frac{\partial a_{66}}{\partial z_{66}}\right)\left(\frac{\partial z_{66}}{\partial a_{33}}\right) \text{ where } \left(\frac{\partial a_{66}}{\partial z_{66}}\right) = \sigma'(z_{66}) = \frac{e^{z_{66}}}{(1+e^{z_{66}})^2} \;\&\; \left(\frac{\partial z_{66}}{\partial a_{33}}\right) = w_{63}'' \qquad \text{Eqn 6.10d}$$

$$\left(\frac{\partial C}{\partial a_{33}}\right) = \left(\frac{\partial C}{\partial a_{33}}\right)_A + \left(\frac{\partial C}{\partial a_{33}}\right)_B + \left(\frac{\partial C}{\partial a_{33}}\right)_C + \left(\frac{\partial C}{\partial a_{33}}\right)_D \qquad \text{Eqn 6.10e}$$

Stage 4: The overall cost function on inputs 1, 2 and 3

The following modifications are made to Eqns 5.0a – 5.6d to address the cost optimization function that pertains to output signal 7 in Fig 4.

$$\left(\frac{\partial C}{\partial w_{30}''}\right) = \left(\frac{\partial C}{\partial a_{33}}\right)\left(\frac{\partial a_{33}}{\partial z_{33}}\right)\left(\frac{\partial z_{33}}{\partial w_{30}''}\right) \text{ where } \left(\frac{\partial z_{33}}{\partial w_{30}''}\right) = x_1 \;\&\; \left(\frac{\partial a_{33}}{\partial z_{33}}\right) = \sigma'(z_{33}) = \frac{e^{z_{33}}}{(1+e^{z_{33}})^2} \qquad \text{Eqn 7.0a}$$

$$\left(\frac{\partial C}{\partial w''_{31}}\right)=\left(\frac{\partial C}{\partial a_{33}}\right)\left(\frac{\partial a_{33}}{\partial z_{33}}\right)\left(\frac{\partial z_{33}}{\partial w''_{31}}\right) \text{ where } \left(\frac{\partial z_{33}}{\partial w''_{31}}\right)=\boldsymbol{x_2} \qquad \text{Eqn 7.0b}$$

$$\left(\frac{\partial C}{\partial w''_{32}}\right)=\left(\frac{\partial C}{\partial a_{33}}\right)\left(\frac{\partial a_{33}}{\partial z_{33}}\right)\left(\frac{\partial z_{33}}{\partial w''_{32}}\right) \text{ where } \left(\frac{\partial z_{33}}{\partial w''_{32}}\right)=\boldsymbol{x_3} \qquad \text{Eqn 7.0c}$$

$$\left(\frac{\partial C}{\partial b_{33}}\right)=\left(\frac{\partial C}{\partial a_{33}}\right)\left(\frac{\partial a_{33}}{\partial z_{33}}\right)\left(\frac{\partial z_{33}}{\partial b_{33}}\right) \text{ where } \left(\frac{\partial z_{33}}{\partial b_{33}}\right)=\boldsymbol{1} \qquad \text{Eqn 7.0d}$$

Hence: $\Delta \boldsymbol{w''_{30}} = -\boldsymbol{R_L}\left(\sum_{i=1}^{n}\left(\frac{\partial C}{\partial w''_{30}}\right)\right)/\boldsymbol{n}$ & $\boldsymbol{w''_{30(New)}} = \boldsymbol{w''_{30(Old)}} + \Delta\boldsymbol{w''_{30}}$ Eqn 7.1a

$$\Delta \boldsymbol{w''_{31}} = -\boldsymbol{R_L}\left(\sum_{i=1}^{n}\left(\frac{\partial C}{\partial w''_{31}}\right)\right)/\boldsymbol{n} \quad \& \quad \boldsymbol{w''_{31(New)}} = \boldsymbol{w''_{31(Old)}} + \Delta\boldsymbol{w''_{31}} \qquad \text{Eqn 7.1b}$$

$$\Delta \boldsymbol{w''_{32}} = -\boldsymbol{R_L}\left(\sum_{i=1}^{n}\left(\frac{\partial C}{\partial w''_{32}}\right)\right)/\boldsymbol{n} \quad \& \quad \boldsymbol{w''_{32(New)}} = \boldsymbol{w''_{32(Old)}} + \Delta\boldsymbol{w''_{32}} \qquad \text{Eqn 7.1c}$$

$$\Delta \boldsymbol{b_{33}} = -\boldsymbol{R_L}\left(\sum_{i=1}^{n}\left(\frac{\partial C}{\partial b_{33}}\right)\right)/\boldsymbol{n} \quad \& \quad \boldsymbol{b_{33(New)}} = \boldsymbol{b_{33(Old)}} + \Delta\boldsymbol{b_{33}} \qquad \text{Eqn 7.1d}$$

Thus, equations 7.2 provide a summary of the formulated learning algorithms for optimizing the overall cost function of the training process for the reconstructed DNN framework in Fig 4.

Stage 1: The overall cost function on output 8

$$\left.\begin{array}{l}
\left(\frac{\partial C}{\partial a_7}\right);\left(\frac{\partial a_7}{\partial z_7}\right);\left(\frac{\partial z_7}{\partial b_7}\right),\left(\frac{\partial z_7}{\partial w_{19}}\right),\left(\frac{\partial z_7}{\partial w_{20}}\right),\left(\frac{\partial \boldsymbol{z_7}}{\partial \boldsymbol{w''_{21}}}\right)\left(\frac{\partial z_7}{\partial w_{21}}\right); \\
\left(\frac{\partial C}{\partial b_7}\right),\left(\frac{\partial C}{\partial w_{19}}\right),\left(\frac{\partial C}{\partial w_{20}}\right),\left(\frac{\partial C}{\partial w_{21}}\right),\left(\frac{\partial \boldsymbol{C}}{\partial \boldsymbol{w''_{21}}}\right)
\end{array}\right\} \qquad \text{Eqn 7.2}$$

Stage 2: The overall cost function on hidden layer 2

$$\left.\begin{array}{l}
\left(\frac{\partial z_7}{\partial a_4}\right),\left(\frac{\partial z_7}{\partial a_5}\right),\left(\frac{\partial z_7}{\partial a_6}\right),\left(\frac{\partial \boldsymbol{z_7}}{\partial \boldsymbol{a_{66}}}\right);\left(\frac{\partial C}{\partial a_4}\right),\left(\frac{\partial C}{\partial a_5}\right),\left(\frac{\partial C}{\partial a_6}\right),\left(\frac{\partial \boldsymbol{C}}{\partial \boldsymbol{a_{66}}}\right); \\
\left(\frac{\partial z_4}{\partial b_4}\right),\left(\frac{\partial z_4}{\partial w_{10}}\right),\left(\frac{\partial z_4}{\partial w_{11}}\right),\left(\frac{\partial z_4}{\partial w_{12}}\right),\left(\frac{\partial \boldsymbol{z_4}}{\partial \boldsymbol{w''_{12}}}\right);\left(\frac{\partial a_4}{\partial z_4}\right);\left(\frac{\partial C}{\partial b_4}\right),\left(\frac{\partial C}{\partial w_{10}}\right),\left(\frac{\partial C}{\partial w_{11}}\right),\left(\frac{\partial C}{\partial w_{12}}\right),\left(\frac{\partial \boldsymbol{C}}{\partial \boldsymbol{w''_{12}}}\right); \\
\left(\frac{\partial z_5}{\partial b_5}\right),\left(\frac{\partial z_5}{\partial w_{13}}\right),\left(\frac{\partial z_5}{\partial w_{14}}\right),\left(\frac{\partial z_5}{\partial w_{15}}\right),\left(\frac{\partial \boldsymbol{z_5}}{\partial \boldsymbol{w''_{15}}}\right);\left(\frac{\partial a_5}{\partial z_5}\right);\left(\frac{\partial C}{\partial b_5}\right),\left(\frac{\partial C}{\partial w_{13}}\right),\left(\frac{\partial C}{\partial w_{14}}\right),\left(\frac{\partial C}{\partial w_{15}}\right),\left(\frac{\partial \boldsymbol{C}}{\partial \boldsymbol{w''_{15}}}\right); \\
\left(\frac{\partial z_6}{\partial b_6}\right),\left(\frac{\partial z_6}{\partial w_{16}}\right),\left(\frac{\partial z_6}{\partial w_{17}}\right),\left(\frac{\partial z_6}{\partial w_{18}}\right),\left(\frac{\partial \boldsymbol{z_6}}{\partial \boldsymbol{w''_{18}}}\right);\left(\frac{\partial a_6}{\partial z_6}\right);\left(\frac{\partial C}{\partial b_6}\right),\left(\frac{\partial C}{\partial w_{16}}\right),\left(\frac{\partial C}{\partial w_{17}}\right),\left(\frac{\partial C}{\partial w_{18}}\right),\left(\frac{\partial \boldsymbol{C}}{\partial \boldsymbol{w''_{18}}}\right); \\
\left(\frac{\partial \boldsymbol{z_{66}}}{\partial \boldsymbol{b_{66}}}\right),\left(\frac{\partial \boldsymbol{z_{66}}}{\partial \boldsymbol{w''_{60}}}\right),\left(\frac{\partial \boldsymbol{z_{66}}}{\partial \boldsymbol{w''_{61}}}\right),\left(\frac{\partial \boldsymbol{z_{66}}}{\partial \boldsymbol{w''_{62}}}\right),\left(\frac{\partial \boldsymbol{z_{66}}}{\partial \boldsymbol{w''_{63}}}\right);\left(\frac{\partial \boldsymbol{a_{66}}}{\partial \boldsymbol{z_{66}}}\right);\left(\frac{\partial \boldsymbol{C}}{\partial \boldsymbol{b_{66}}}\right),\left(\frac{\partial \boldsymbol{C}}{\partial \boldsymbol{w''_{60}}}\right),\left(\frac{\partial \boldsymbol{C}}{\partial \boldsymbol{w''_{61}}}\right),\left(\frac{\partial \boldsymbol{C}}{\partial \boldsymbol{w''_{62}}}\right),\left(\frac{\partial \boldsymbol{C}}{\partial \boldsymbol{w''_{63}}}\right)
\end{array}\right\} \qquad \text{Eqn 7.3}$$

Stage 3: The overall cost function on hidden layer 1

$$\left.\begin{array}{l}
\left(\frac{\partial z_4}{\partial a_1}\right),\left(\frac{\partial z_5}{\partial a_1}\right),\left(\frac{\partial z_6}{\partial a_1}\right),\left(\frac{\partial \boldsymbol{z_{66}}}{\partial \boldsymbol{a_1}}\right);\left(\frac{\partial C}{\partial a_1}\right)_A,\left(\frac{\partial C}{\partial a_1}\right)_B,\left(\frac{\partial C}{\partial a_1}\right)_C,\left(\frac{\partial \boldsymbol{C}}{\partial \boldsymbol{a_1}}\right)_{\boldsymbol{D}};\left(\frac{\partial C}{\partial a_1}\right); \\
\left(\frac{\partial z_4}{\partial a_2}\right),\left(\frac{\partial z_5}{\partial a_2}\right),\left(\frac{\partial z_6}{\partial a_2}\right),\left(\frac{\partial \boldsymbol{z_{66}}}{\partial \boldsymbol{a_2}}\right);\left(\frac{\partial C}{\partial a_2}\right)_A,\left(\frac{\partial C}{\partial a_2}\right)_B,\left(\frac{\partial C}{\partial a_2}\right)_C,\left(\frac{\partial \boldsymbol{C}}{\partial \boldsymbol{a_2}}\right)_{\boldsymbol{D}};\left(\frac{\partial C}{\partial a_2}\right); \\
\left(\frac{\partial z_4}{\partial a_3}\right),\left(\frac{\partial z_5}{\partial a_2}\right),\left(\frac{\partial z_6}{\partial a_3}\right),\left(\frac{\partial \boldsymbol{z_{66}}}{\partial \boldsymbol{a_3}}\right);\left(\frac{\partial C}{\partial a_3}\right)_A,\left(\frac{\partial C}{\partial a_3}\right)_B,\left(\frac{\partial C}{\partial a_3}\right)_C,\left(\frac{\partial \boldsymbol{C}}{\partial \boldsymbol{a_3}}\right)_{\boldsymbol{D}};\left(\frac{\partial C}{\partial a_3}\right);
\end{array}\right\} \qquad \text{Eqn 7.4}$$

$$\left(\frac{\partial z_4}{\partial a_{33}}\right), \left(\frac{\partial z_5}{\partial a_{33}}\right), \left(\frac{\partial z_6}{\partial a_{33}}\right), \left(\frac{\partial z_{66}}{\partial a_{33}}\right); \left(\frac{\partial C}{\partial a_{33}}\right)_A, \left(\frac{\partial C}{\partial a_{33}}\right)_B, \left(\frac{\partial C}{\partial a_{33}}\right)_C, \left(\frac{\partial C}{\partial a_{33}}\right)_D; \left(\frac{\partial C}{\partial a_{33}}\right)$$

Stage 4: The overall cost function on inputs 1, 2 and 3

$$\left.\begin{array}{l}
\left(\frac{\partial z_1}{\partial b_1}\right), \left(\frac{\partial z_1}{\partial w_1}\right), \left(\frac{\partial z_1}{\partial w_2}\right), \left(\frac{\partial z_1}{\partial w_3}\right); \left(\frac{\partial a_1}{\partial z_1}\right), \left(\frac{\partial C}{\partial a_1}\right); \left(\frac{\partial C}{\partial b_1}\right), \left(\frac{\partial C}{\partial w_1}\right), \left(\frac{\partial C}{\partial w_2}\right), \left(\frac{\partial C}{\partial w_3}\right); \\
\left(\frac{\partial z_2}{\partial b_2}\right), \left(\frac{\partial z_2}{\partial w_4}\right), \left(\frac{\partial z_2}{\partial w_5}\right), \left(\frac{\partial z_2}{\partial w_6}\right); \left(\frac{\partial a_2}{\partial z_2}\right), \left(\frac{\partial C}{\partial a_2}\right); \left(\frac{\partial C}{\partial b_2}\right), \left(\frac{\partial C}{\partial w_4}\right), \left(\frac{\partial C}{\partial w_5}\right), \left(\frac{\partial C}{\partial w_6}\right); \\
\left(\frac{\partial z_3}{\partial b_3}\right), \left(\frac{\partial z_3}{\partial w_7}\right), \left(\frac{\partial z_3}{\partial w_8}\right), \left(\frac{\partial z_3}{\partial w_9}\right); \left(\frac{\partial a_3}{\partial z_3}\right), \left(\frac{\partial C}{\partial a_3}\right); \left(\frac{\partial C}{\partial b_3}\right), \left(\frac{\partial C}{\partial w_7}\right), \left(\frac{\partial C}{\partial w_8}\right), \left(\frac{\partial C}{\partial w_9}\right); \\
\left(\frac{\partial z_{33}}{\partial b_{33}}\right), \left(\frac{\partial z_{33}}{\partial w''_{30}}\right), \left(\frac{\partial z_{33}}{\partial w''_{31}}\right), \left(\frac{\partial z_{33}}{\partial w''_{32}}\right); \left(\frac{\partial a_{33}}{\partial z_{33}}\right); \left(\frac{\partial C}{\partial b_{33}}\right), \left(\frac{\partial C}{\partial w''_{30}}\right), \left(\frac{\partial C}{\partial w''_{31}}\right), \left(\frac{\partial C}{\partial w''_{32}}\right);
\end{array}\right\} \quad \text{Eqn 7.5}$$
